# A taxonomic revision of *Cymbopogon* (Poaceae) in Thailand

**DOI:** 10.7717/peerj.21297

**Published:** 2026-05-18

**Authors:** Paweena Wessapak, Chatchai Ngernsaengsaruay, Sutee Duangjai

**Affiliations:** 1 Department of Royal Rainmaking and Agricultural Aviation, Chatuchak, Bangkok, Thailand; 2 Department of Botany, Faculty of Science, Kasetsart University, Chatuchak, Bangkok, Thailand; 3 Biodiversity Center Kasetsart University (BDCKU), Chatuchak, Bangkok, Thailand; 4 Department of Forest Biology, Faculty of Forestry, Kasetsart University, Chatuchak, Bangkok, Thailand

**Keywords:** Andropogoneae, Anthistiriinae, C4 grass, Essential oil, Gramineae, Lectotype, Taxonomy

## Abstract

The genus *Cymbopogon* (Poaceae) in Thailand is revised. Twelve species are recognized, including *C. calciphilus*, endemic to Thailand. Three species, *C. citratus*, *C. nardus*, and *C. winterianus*, are widely cultivated, considered introduced, and economically important for their aromatic oils, which are used in traditional medicine, perfumery, and other industries; however, there is no evidence that they have become naturalized in the wild. Detailed morphological descriptions, illustrations, and photographs are provided, along with notes on distribution, habitat and ecology, phenology, vernacular names, and uses. Specimens examined are thoroughly documented. Three names are lectotypified in this study: *C. cambogiensis*, *C. khasianus*, and *C. confertiflorus*, which is a synonym of *C. nardus*. This study provides an updated taxonomic framework for the genus in Thailand and clarifies nomenclatural issues.

## Introduction

Poaceae Barnhart (Gramineae Juss., nom. alt.) is one of the largest families of flowering plants, comprising 11,783 species in 789 accepted genera worldwide (Soreng et al., 2022). The family occurs across a wide range of habitats and tolerates diverse environmental stresses. Grasses are of major economic importance, as they provide key staple food crops for humans, including rice (*Oryza sativa*), wheat (*Triticum aestivum*), oats (*Avena sativa*), and maize (*Zea mays*), which are essential to global food security (Chase, 1921; Bor, 1960; Gould, 1968). Many grass species are also cultivated as forage and fodder crops for livestock (Oyen & Dung, 1999; Obi Reddy et al., 2014). In addition, some grasses are important sources of essential oils and aromatic compounds, notably species of *Cymbopogon* Spreng., which are widely used in the cosmetic and pharmaceutical industries and as flavouring agents in food (Bor, 1960; Chavre & Sonawane, 2021).

The classification of the grass family has traditionally been based on morphological and anatomical characters. In recent decades, molecular DNA data have been increasingly applied to clarify phylogenetic relationships within the family, resulting in substantial improvements in the understanding of its evolutionary history (Mathews, Tsai & Kellogg, 2000; Kellogg, 2015; Soreng et al., 2015; Soreng et al., 2017). According to the most recent classification, Poaceae is divided into 12 subfamilies, 54 tribes, and 109 subtribes. Among these, Pooideae Benth. is the most species-rich subfamily, comprising 4,126 species in 219 genera, followed by Panicoideae A. Braun with 3,325 species in 242 genera, representing the second-largest subfamily by species richness (Soreng et al., 2022). Eleven genera are placed in the subtribe Anthistiriinae in the updated worldwide phylogenetic classification of Poaceae; eight of these genera are represented in Thailand, namely *Bothriochloa*, *Capillipedium*, *Cymbopogon*, *Dichanthium*, *Heteropogon*, *Iseilema*, *Pseudanthistiria*, and *Themeda* (Soreng et al., 2022). This classification is consistent with phylogenomic evidence supporting the tribe Andropogoneae (Welker et al., 2020).

*Cymbopogon*, a C_4_ grass genus (Watson & Dallwitz, 1994; Soreng et al., 2015), belongs to the subfamily Panicoideae, tribe Andropogoneae Dumort., and subtribe Anthistiriinae J. Presl (Soreng et al., 2015; Soreng et al., 2022). The genus was traditionally placed in the subtribe Andropogoninae by Clayton & Renvoize (1986), Kellogg (2015), and Soreng et al. (2017). However, recent phylogenetic studies, particularly by Soreng et al. (2015); Soreng et al. (2022), have clarified the relationships within the Andropogoneae and strongly supported the placement of *Cymbopogon* in the subtribe Anthistiriinae. Molecular phylogenetic analyses based on DNA sequence data (such as the studies by Soreng et al., 2017; Soreng et al., 2022) have revealed distinct lineages within Anthistiriinae, with *Cymbopogon* forming a well-supported clade that is more closely related to genera such as *Bothriochloa* and *Themeda*, than to the subtribe Andropogoninae. These studies have thus provided robust evidence for the reclassification of *Cymbopogon* into Anthistiriinae, marking a significant advancement in the understanding of its phylogenetic positioning within Andropogoneae. Most species of *Cymbopogon* can be readily distinguished from related genera within the tribe by their characteristic aromatic scent (Soenarko, 1977).

The genus *Cymbopogon* comprises mostly perennial, tufted grasses with mainly aromatic leaf blades and a membranous ligule. Its synflorescence is compound and bears paired racemes subtended by boat-shaped spatheoles, forming a false panicle. Spikelets are found as paired sessile and pedicelled spikelets, and florets have two lodicules and three stamens (Clayton & Renvoize, 1986; Duistermaat, 2005; Chen & Phillips, 2006; Clayton et al., 2006). The genus was taxonomically revised by Soenarko (1977), who recognized 55 species, whereas more recent compilations record 59 species (Soreng et al., 2022), with 54 species currently accepted in Plants of the World Online (POWO, 2025). *Cymbopogon* is mainly distributed throughout the Old World tropics and subtropics (Soenarko, 1977; POWO, 2025), occurring in Africa, Asia, Australasia, and the Pacific, with some species also present in the Americas (Clayton et al., 2006). Several species are of considerable economic importance and are widely used as sources of essential oils, as well as in culinary and medicinal applications. Nevertheless, most species remain poorly studied and are often overlooked in favor of a few well-known taxa, particularly *C. citratus* (DC.) Stapf and *C. nardus* (L.) Rendle, which dominate both research attention and commercial use.

In Asia, the distribution of *Cymbopogon* has been documented in several regional floristic treatments. In the Indian subcontinent, including India, Sri Lanka, Myanmar, and Pakistan, 23 species were reported (Bor, 1960). In China, the genus is represented by 20 native species, with an additional four species recorded in cultivation (Chen & Phillips, 2006). In Peninsular Malaysia, five species were reported, comprising two wild and three cultivated species (Gilliland, 1971). In Singapore, five species were recorded, all of which are non-native and cultivated for the production of aromatic essential oils (Duistermaat, 2005). In Java, the genus was recorded as comprising one wild species and five cultivated species (Backer & Bakhuizen van den Brink, 1968).

In Thailand, the only comprehensive treatment of *Cymbopogon* was conducted by Nanakorn (1990) in an unpublished Ph.D. dissertation, in which the genus was placed in the subtribe Andropogoninae and reported to comprise 13 species for the Thai flora. Since this work remains unpublished and has not been updated in the regional floristic literature, a current revision of *Cymbopogon* in Thailand is necessary to formally document its species diversity. In the present paper, we provide an updated account of *Cymbopogon* in Thailand, presenting a comprehensive taxonomic treatment that includes lectotypifications, morphological descriptions, illustrations, and photographs, together with notes on distribution, habitat and ecology, phenology, vernacular names, uses, and specimens examined. An identification key to the species of *Cymbopogon* in Thailand is also provided.

## Materials & Methods

Thailand’s floristic regions follow *Flora of Thailand* Vol. 16(4) (The Forest Herbarium, Department of National Parks, Wildlife and Plant Conservation, 2025). *Cymbopogon* species were surveyed and specimens were collected across all floristic regions of Thailand. Collected specimens were examined through a comprehensive review of the literature (*e.g.*, Bor, 1953; Bor, 1954; Bor, 1960; Bor, 1965; Backer & Bakhuizen van den Brink, 1968; Gilliland, 1971; Soenarko, 1977; Clayton & Renvoize, 1982; Nanakorn, 1990; Duistermaat, 2005; Chen & Phillips, 2006; Turner et al., 2019; Veldkamp et al., 2019) and by comparison with herbarium specimens housed in the Aarhus University Herbarium (AAU), Bangkok Herbarium (BK), Department of National Parks, Wildlife and Plant Conservation (BKF), The Natural History Museum (BM), University of Copenhagen (C), the Royal Botanic Gardens (K), Muséum National d’Histoire Naturelle (P), Prince of Songkla University (PSU), Queen Sirikit Botanic Garden, The Botanical Garden Organization (QBG), as well as those accessible through virtual herbarium databases, including BM, Royal Botanic Garden Edinburgh (E), University of Florence, University Museum System, Museum of Natural History (FI), Naturalis Biodiversity Center (L), the Swedish Museum of Natural History (S), the Singapore Herbarium (SING), Naturhistorisches Museum Wien (W), and the Smithsonian Institution (US), and *via* GBIF Secretariat (2023). Herbarium acronyms follow Thiers (2025). The taxonomic history of each species was compiled from both published literature and online databases (*e.g.*, Bor, 1953; Gilliland, 1971; Soenarko, 1977; Clayton & Renvoize, 1982; Duistermaat, 2005; Chen & Phillips, 2006; Veldkamp et al., 2019; IPNI, 2025; POWO, 2025; World Flora Online (WFO), 2025). Morphological characters, distributions, habitats, ecology, phenology, and uses were documented from both historic and newly collected specimens, as well as from field observations by the authors. Vernacular names were compiled from the specimens examined and relevant literature (*e.g.*, Pooma & Suddee, 2014). To determine whether the *Cymbopogon* species are native or introduced in Thailand, we reviewed historical records, including herbarium specimens and literature. We cross-referenced species distribution data with known native ranges and identified occurrences of these species in Thailand. For species reported as non-native or introduced, we used records of plant introductions to confirm their status and ensure they have not spread into natural forests.

## Results

### Taxonomic treatment

***Cymbopogon*** Spreng., Pl. Min. Cogn. Pug. 2: 14. 1815; Ridl., Fl. Malay. Penins. 5: 211. 1925; Bor, Grasses Burma, Ceyl. Ind. & Pakist.: 121. 1960; Ohwi, Fl. Jap., revised ed.: 192. 1965; Backer & Bakh. f., Fl. Java 3: 610. 1968; Gilliland, Re. Fl. Mal. 3: 294. 1971; Soenarko, Reinwardtia 9: 273. 1977; Clayton & Renvoize, Gen. Gram.: 351. 1986; Duistermaat, Gard. Bull. Singapore 57: 43. 2005; S. L. Chen & S. M. Phillips in C. Y. Wu, P. H. Raven & H. Deyuan, Fl. China 22: 624. 2006.

≡*Andropogon* L. sect. *Cymbopogon* (Spreng.) Steud., Syn. PI. Glum. 1: 383. 1854. Type: *Andropogon schoenanthus* L. ≡ *Cymbopogon schoenanthus* (L.) Spreng.

= *Gymnanthelia* Andersson in Schweinf., Beitr. Fl. Aethiop. 1: 299. 1867, *nom. nud.*

= *Andropogon* L. subgen. *Cymbopogon* Nees sect. *Gymnanthelia* Hackel in DC, Monogr. Phan. 6: 594. 1889. Type: not known.

Mostly aromatic and perennial grasses. *Culms* erect. *Leaf blades* linear, usually glabrous. *Ligules* chartaceous. *Synflorescence* broad (usually more than 15 cm width) or narrow (usually less than 15 cm width). *True inflorescences* consist of paired racemes, each pair subtended by a spatheole, and enclosed at the base by surrounding spatheole bracts. *Racemes* slender (usually more than two cm long) or short (less than two cm long); raceme-bases flattened or swollen, unequal in length; rachis fragile at the node; rachis internode tip cupuliform or truncate. *Spikelets* in pair (one sessile and one pedicelled) or triad (one sessile and two pedicelled) at terminal raceme; homogamous spikelet pairs present at base of lower raceme; upper raceme without homogamous pair. *Lower glume* winged or wingless, with or without median groove. *Upper glume* usually keeled, with or without winged on keel. *Florets* 2. *Lower floret* male or sterile. *Upper floret* bisexual; upper lemma hyaline, with distinct, shortly exserted awn or awnless. *Lodicules* 2. *Stamens* 3.

**Figure 1:**
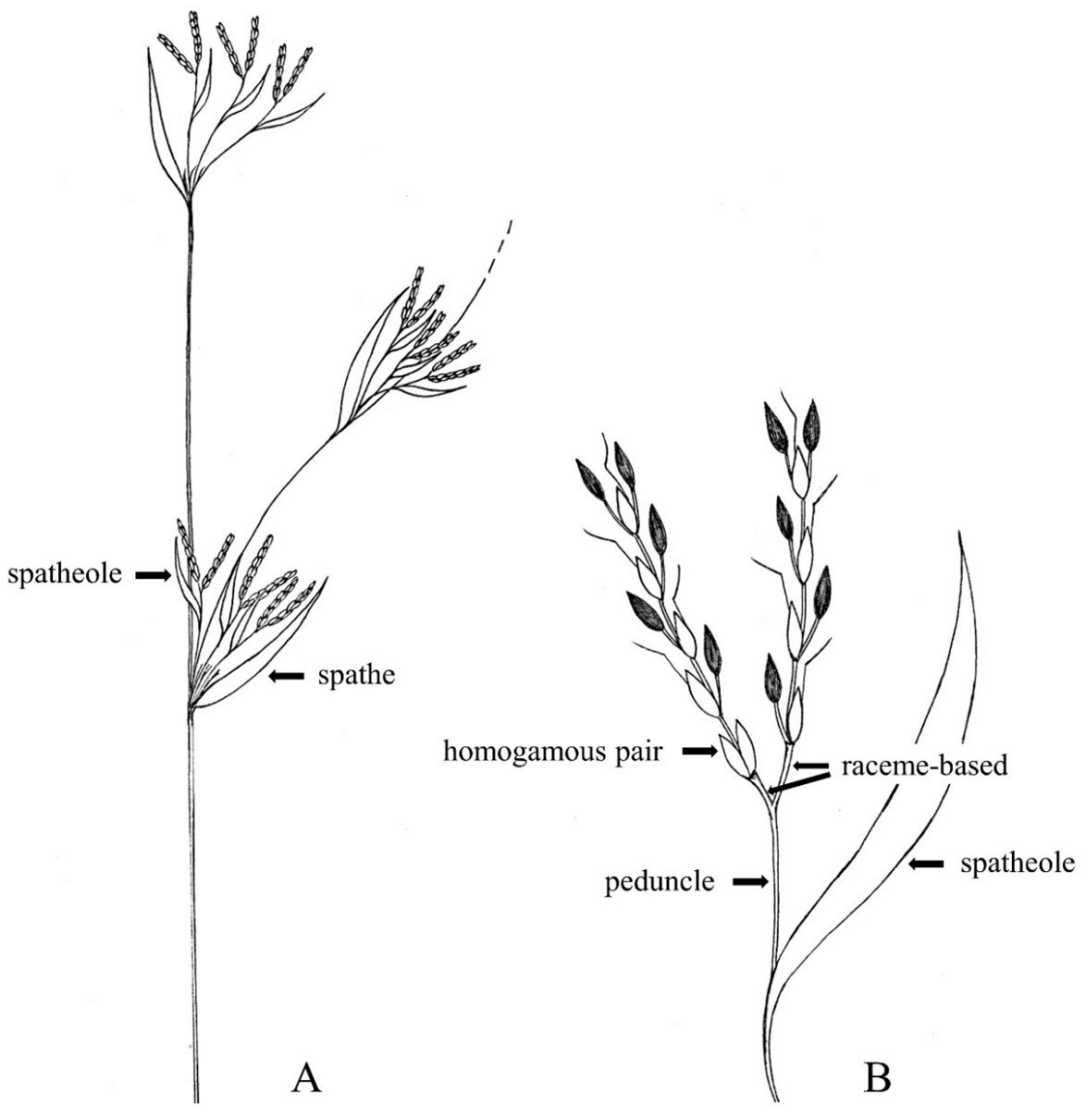
Part of synflorescence of the genus *Cymbopogon*. (A) Part of synflorescence. (B) True inflorescence, which in this study refers specifically to a unit consisting of a pair of racemes subtended by a spatheole. Photo: Drawn by Paweena Wessapak.

In *Cymbopogon*, the inflorescence is a synflorescence of the spatheate panicle type. A single synflorescence is usually terminal on a culm and occasionally axillary. The term synflorescence here refers to a compound spatheate panicle in which each branch is subtended by a spathe. These spathes initially resemble leaf sheaths with reduced blades. The most modified of these bracts, which subtends the true inflorescence, is termed the spatheole, and it supports a pair of racemes (Soenarko, 1977) (Fig. 1A).

In this study, the phrase “true inflorescence” refers specifically to a unit consisting of a pair of racemes subtended by a spatheole. This unit corresponds to the ray *sensu* Soenarko (1977), and functions as a single branching unit within the synflorescence. The synflorescence represents a spatheate panicle composed of multiple such units (tiers *sensu* Soenarko) (Fig. 1B). The racemes of *Cymbopogon* are spike-like, composed of paired sessile and pedicelled spikelets arranged along the rachis. Each pair of racemes arises from a stalk (raceme base) that is unequal or subequal, usually flattened and often deflexed at maturity. The spatheoles in the genus are consistently narrow and boat-shaped, typically glabrous or nearly so, although some species (*e.g.*, *C. microstachys*) may show slight pubescence near the apex. Although the length of the spatheole varies among species, it is generally longer than the filiform peduncle; however, this variation is not considered a key taxonomic character for the genus.

This genus is usually aromatic grass comprising of 54 species worldwide (POWO, 2025), out of which 12 species are presented in Thailand (including the species that introduced and widely cultivated to useful in the country). Three species, *i.e.,* *Cymbopogon citratus*, *C. nardus*, and *C. winterianus*, have all been recorded as being in cultivation but there is no evidence that become naturalized in Thailand.

### A key to the species of *Cymbopogon* in Thailand

**Table:** 

1a. Lower glume of sessile spikelet with a median groove from the middle downwards ……. . . 2
1b. Lower glume of sessile spikelet without a median groove from the middle downwards, flat, wrinkle on the back or slightly concave at the base. ………………………………………. . . 4
2a. Sessile spikelet usually awnless or awn shortly exserted; lower glume 2.5–3.2 mm long with a shallow median groove; the lowermost pedicel somewhat flattened…..……** 4.*C. cambogiensis***
2b. Sessile spikelet with a distinct awn; lower glume more than 3.2 mm long with a deep median groove; the lowermost pedicel swollen………………………………………………………3
3a. Plants 0.7–1.5(−1.8) m high; leaf blade 0.2–0.6 cm wide; base narrow, attenuate or slightly rounded………………………………………...…………………………** 1.*C. annamensis***
3b. Plants 1.5–2 m high; leaf blade 0.8–1.5 cm wide; base cordate, often amplexicaul…………... ……………………………………….……………..…………………………** 8.*C. martini***
4a. Most of leaf blade less than 1.4 cm wide (sometimes up to 1.5 cm wide in *C. traninhensis* but its sessile spikelet usually more than 6.5 mm long); synflorescence narrow, usually less than 15 cm in width…………………………………………………………..…………..………5
4b. Most of leaf blade more than 1.4 cm wide; synflorescence broad, usually more than 15 cm width……...…………………………..………………………...………………………….. 9
5a. Lower glume of sessile spikelet narrowly winged or wingless, usually less than five mm long …………………………………………………………………………………………….. 6
5b. Lower glume of sessile spikelet broadly winged, usually more than five mm long…………. 8
6a. Basal sheath and nodes usually tomentose, rarely glabrescent; collar and triangular patches at the tip of leaf sheath hairy......................................................................................... ** 3.*C. calciphilus***
6b. Basal sheath, nodes, and triangular patches at the tip of leaf sheath usually glabrous or glabrescent; collar glabrous or sometimes tomentose…………………………………………. 7
7a. Raceme slender, 2–2.5 cm long; pedicel and rachis internode 2.5–3.3 mm long; leaf blade 4–7(–10) mm wide………………………………………………………………. ** 2.*C. calcicola***
7b. Raceme short, 0.9–1.5 cm long; pedicel and rachis internode 2–2.5 mm long; leaf blade 1–1.4 cm wide...................................................................................................................** 9.*C. microstachys***
8a. Lower glume of sessile spikelet oblong-lanceolate, 5.2–6 mm long, usually 4- or 5-nerved .................................................................................................................................... ** 7.*C. khasianus***
8b. Lower glume of sessile spikelet lanceolate or broadly lanceolate, (6–)6.5–8.5 mm long, 2-nerved or nerveless............................................................................................ ** 11.*C. traninhensis***
9a. Sessile spikelet with a distinct awn, awn 1–1.5 cm long; at the base of leaf blade with long hairs on upper surface.......................................................................................................... ** 6.*C. flexuosus***
9b. Sessile spikelet awnless, awn reduced to a bristle or very slender awn, less than 1 cm long; at the base of leaf blade glabrous or glabrescent................................................................................. 10
10a. Lower glume of sessile spikelet, oblong-lanceolate or linear-lanceolate, (5–)6–7.7 long; upper lemma usually awnless; widely cultivated for culinary use......................................... ** 5.*C. citratus***
10b. Lower glume of sessile spikelet lanceolate or narrowly lanceolate, 3.5–5(−5.5) mm long; upper lemma with short to long awn; widely cultivated for citronella oil production..................... 11
11a. Panicle dense or much congested; true inflorescence often interrupted; raceme 1–1.2(−1.5) cm long......................................................................................................................... ** 10.*C. nardus***
11b. Panicle lax; true inflorescence not interrupted, arranged zig-zag; raceme 1.2–2.2 cm long ................................................................................................................................** 12.*C. winterianus***

**1. *Cymbopogon annamensis*** (A. Camus) A. Camus, Bull. Mus. Natl. Hist. Nat. 26: 563. 1920; Soenarko, Reinwardtia 9: 335. 1977.

≡ *Cymbopogon martini* (Roxb.) Will. Watson var. *annamensis* A. Camus, Bull. Mus. Natl. Hist. Nat. 25: 670. 1919. Type: Vietnam, 10 Feb 1914, *A. J. B. Chevalier s.n.* (holotype: **P**! [P01942873]).

= *Cymbopogon bassacensis* A. Camus, Bull. Mus. Natl. Hist. Nat. 26: 564. 1920. Type: Vietnam, Mekong, 1866, *C. Thorel s.n.* (holotype: **P**! [P00745755]).

**Figure 2:**
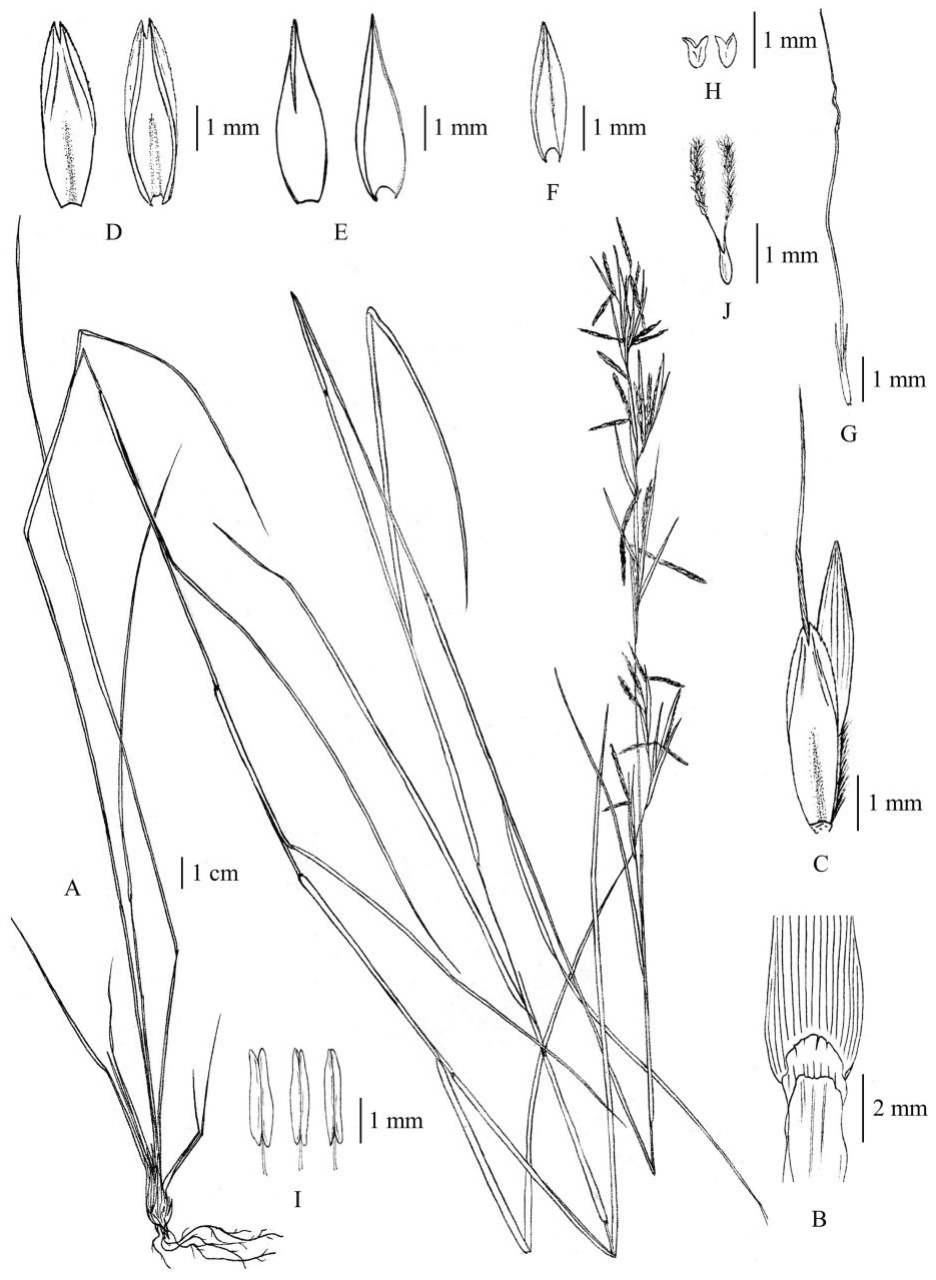
*Cymbopogon annamensis*. (A) Habit. (B) Ligule. (C) Paired spikelet; D: Lower glumes. (E) Upper glumes. (F) Lower lemma. (G) Upper lemma with distinct awn. (H) Lodicules. (I) Stamens. (J) Pistil. Photo: Drawn by Paweena Wessapak.

**Figure 3:**
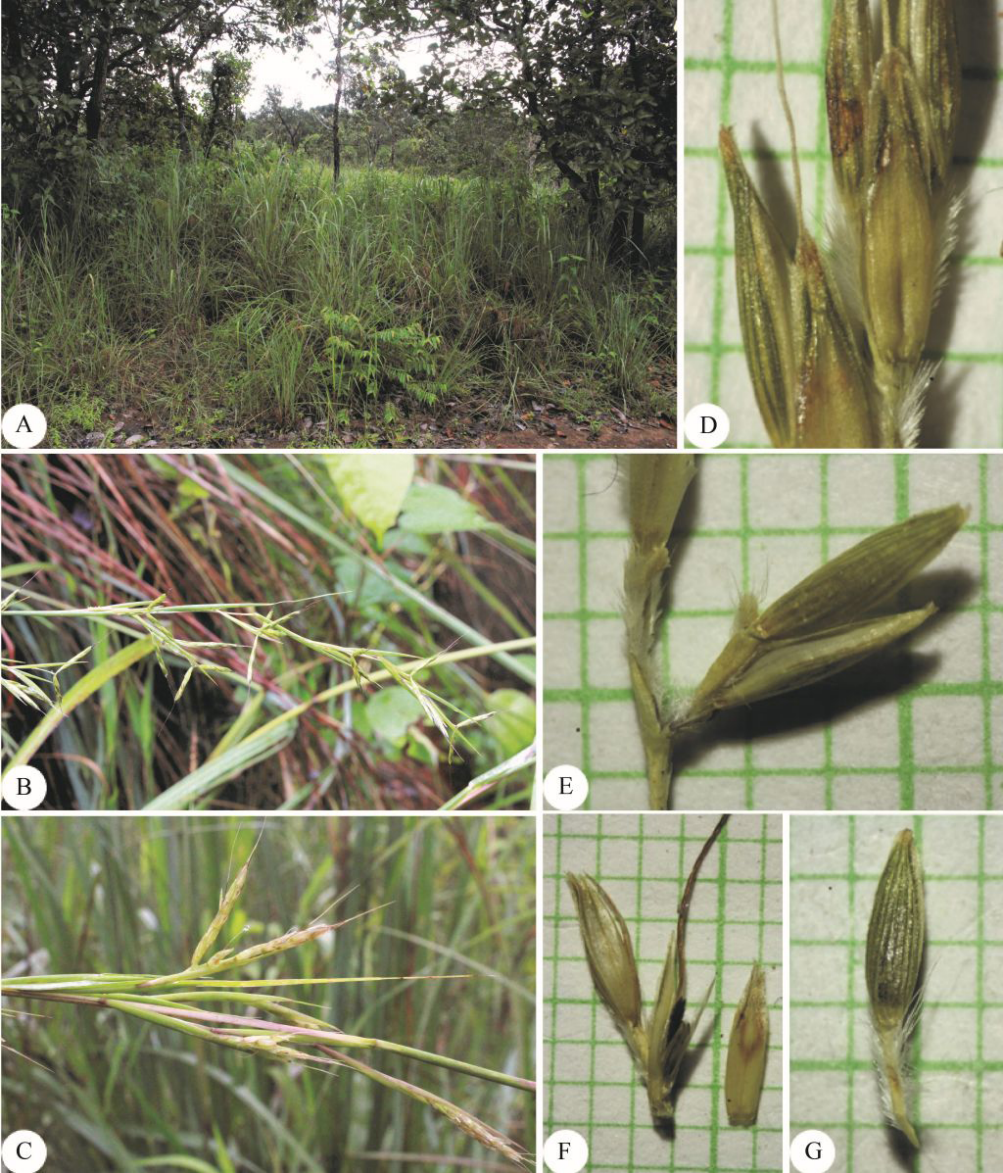
*Cymbopogon annamensis*. (A) Habitat and habit. (B–C) Parts of synflorescences. (D) Spikelets on raceme. (E) Raceme-based. (F) Dissected of sessile spikelet and lower glume separated. (G) Pedicelled spikelet. Photos: Paweena Wessapak.

Perennial, loosely tufted. *Culms* slightly geniculate ascending or erect, slender, 0.7–1.5 (−1.8) m high (including synflorescences); nodes glabrous; internodes terete, 6–25 cm long, 1–3.5 mm diam., glabrous. *Leaf sheaths* 3.8–12 cm long, margins membranous and entire, glabrous. *Ligules* chartaceous, 0.5–1.5 mm long. *Collar* glabrous. *Leaf blade* linear, 10–40 cm × 2–6 mm, apex acute, base narrow attenuate or slightly rounded, margins scabrous, chartaceous, glabrous on both surfaces. *Synflorescence* 15–70 × 2–8 cm.; synflorescence a spatheate panicle composed of multiple true inflorescences; each true inflorescence consisting of a pair of spike-like racemes subtended by a spatheole. *Racemes* 2, subtended by spatheole 1.6–3.8 cm long, chartaceous, glabrous; lower raceme 1.1–1.9 cm long, 4–6 spikelet pairs (including homogamous pair); upper raceme 1.2–2.2 cm long, 4–8 spikelet pairs; raceme-based flattened, hairy along margins; rachis internode flattened, hairy along margins, glabrous on the back. *Pedicel of homogamous pair* swollen. *Peduncle* terete or subterete, 0.6–2.7 cm long, glabrous and slightly hairy at the tip. *Spikelet* in pair (one sessile and one pedicelled) or triad (one sessile and two pedicelled). *Sessile spikelets* oblong-oblanceolate or lanceolate, 3.4–4.8 × 0.8–1 mm. *Lower glume* oblong-oblanceolate or lanceolate, 3.4–4.8 × 0.8–1 mm, apex bifid, broadly winged, margins entire, chartaceous, a deep median groove below the middle of the lower glume, glabrous, 2-nerved or nerveless. *Upper glume* lanceolate, boat-shape, 3.4–4.5 × ca. 0.8 mm, apex acuminate, margins entire to slightly ciliate, chartaceous, 1-keeled with winged, 1-nerved. *Florets* 2. *Lower floret* sterile. *Lower lemma* oblong-lanceolate, 2.5–3.4 × ca. 0.7 mm, apex acute, margins ciliate, hyaline, glabrous, nerveless. *Lower palea* absent. *Upper floret* bisexual. *Upper lemma* 1.5–2 mm long, apex bifid, awn from sinus; awn geniculate, 1.3–1.5 cm long. *Upper palea* absent. *Lodicules* cuneate, ca. 0.5 mm long, truncate. *Stamens*: filament filiform; anther purple or yellowish-purple, 1.5–2 mm long. *Pistil*: ovary ovate in outline, ca. 0.2 × 0.1 mm; style 2; stigma plumose. *Caryopsis* not seen. *Pedicelled spikelets* narrowly elliptic or elliptic-lanceolate, 3.3–4.3 × 0.8–1 mm. *Pedicel* flattened, 2–2.3 mm long, hairy along margins, glabrous on the back. *Lower glume* narrowly elliptic or elliptic-lanceolate, 3.3–4.3 × 0.8–1 mm, apex acute, margins narrowly winged or wingless, subchartaceous, glabrous, 9-nerved. *Upper glume* elliptic-lanceolate, 3.2–4.2 × 0.7–1 mm, apex acute, margins ciliate, chartaceous, glabrous, 1- or 3-nerved. *Lower lemma* lanceolate, 2.8–3.8 × ca. 0.5 mm, apex acute or acuminate, margins ciliate, hyaline, glabrous, nerveless (Figs. 2 & 3).

**Distribution.** China (Yunnan), Vietnam, Laos, and Thailand.

**Distribution in Thailand.** **North-Eastern**: Phetchabun; **Eastern**: Chaiyaphum, Nakhon Ratchasima.

**Habitat and Ecology.** By roadsides, at the base of granite hills, in open grassland, and in open areas of deciduous forest; 200–600 m alt.

**Phenology.** Flowering and fruiting from August to December.

**Vernacular name.** **Ta khrai pa bai khaep** (**
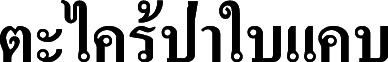
**), proposed here.

**Uses.** Not reported.

**Specimens examined.** **North-Eastern**: Phetchabun [Thung Salaeng Luang, 600 m alt., 27 Aug 2017, *P. Wessapak 383* (BK); ibid., 600 m alt., 30 Sep 2017, *P. Wessapak 402* (BK)]; ibid., 12 Oct 2018, *P. Wessapak, W. Arthan & J. Satthaphorn 479* (BK)]; **Eastern**: Chaiyaphum [Phu Khiao Wildlife Sanctuary, 300 m alt., 11 Nov 1984, *W. Nanakorn et al. 2084* (QBG)]; Nakhon Ratchasima [Locality unspecified, 23 Nov 1923, *A. F. G. Kerr 7948* (BK, K); Pak Thong Chai, 200 m alt., 27 Dec 1923, *A. F. G. Kerr 8130* (AAU, BK, BM, K); Bua Yai, 1 Nov 1931, *Put 4245* (BM, BK, K)].

**2. *Cymbopogon calcicola*** C. E. Hubb., Bull. Misc. Inform. Kew 1941: 24. 1941; Gilliland, Re. Fl. Mal. 3: 297. 1971; Soenarko, Reinwardtia 9: 367. 1977. Type: Peninsular Malaysia, Kedah, Gunong Baling, Jan 1939, *SFN 36256* (holotype: **K**! [K000290043]; isotypes: **K**! [K000290080, K000290081, K000290082, K000290083], **SING** [SING0054766, SING0054767, Herbarium Online]).

**Figure 4:**
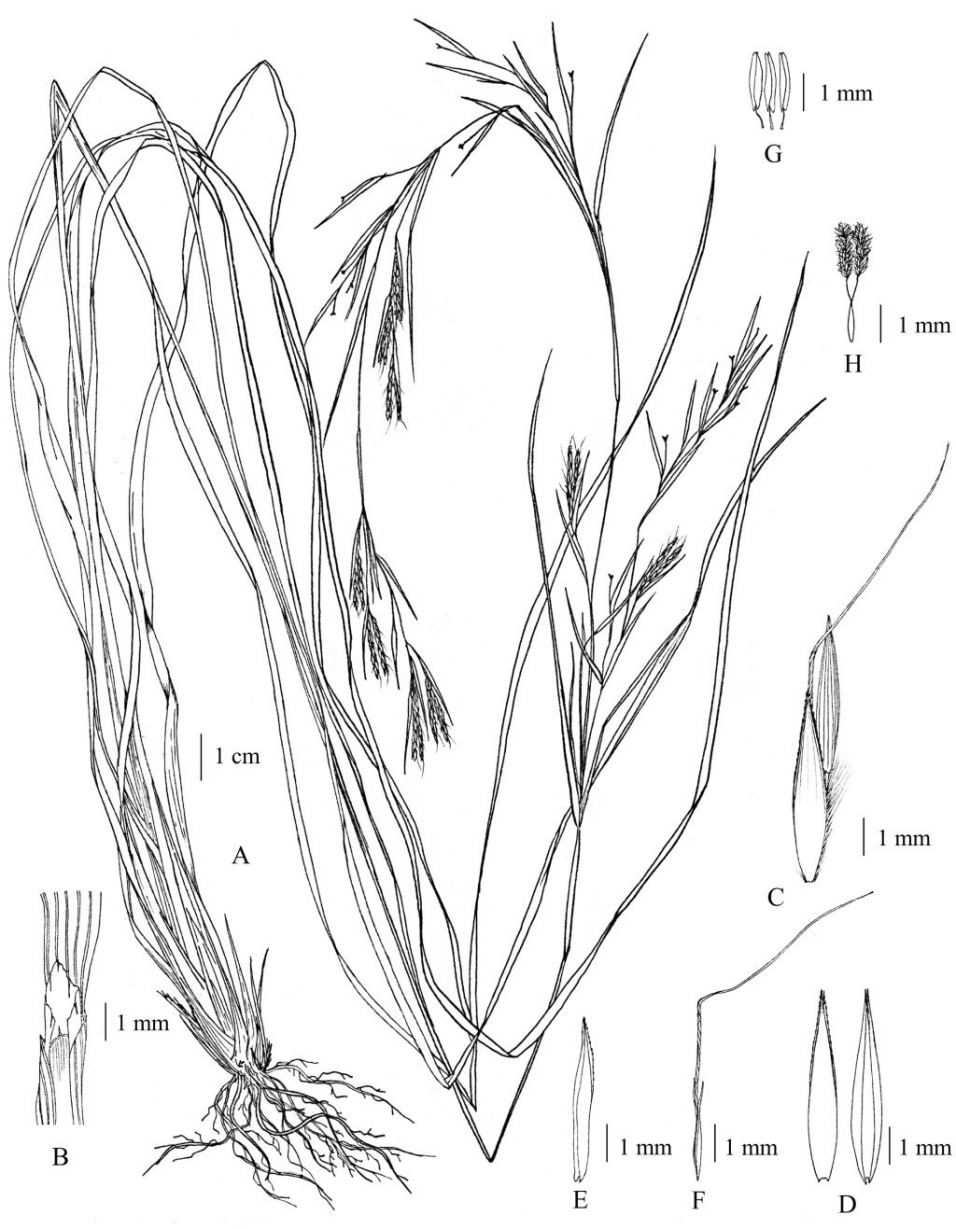
*Cymbopogon calcicola*. (A) Habit. (B) Ligule. (C) Paired spikelet. (D) Lower glumes. (E) Upper glume. (F) Upper lemma with distinct awn. (G) Stamens. (H) Pistil. Photo: Drawn by Paweena Wessapak.

**Figure 5:**
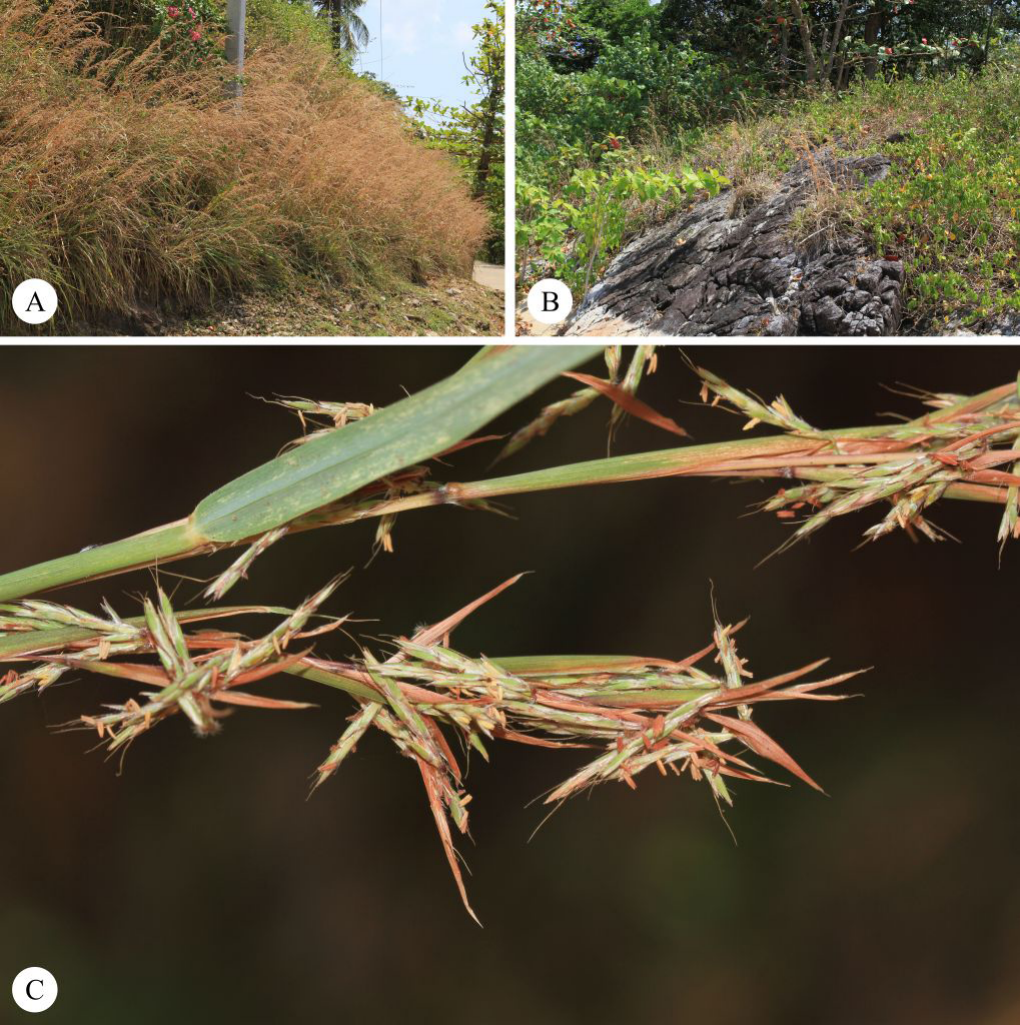
*Cymbopogon calcicola*. (A–B) Habitat and habit. (C) Parts of synflorescence. Photos: Chatchai Ngernsaengsaruay.

Perennial, tufted. *Culms* erect, up to 1.5 m high (including synflorescences); nodes glabrous; internodes terete, 14–22 cm long, 1–2.5 mm diam., glabrous. *Leaf sheaths* 9–21 cm long, base glabrous, margins membranous and entire, glabrous. *Ligules* chartaceous 1.5–5 mm long. *Collar* glabrous. *Leaf blades* linear, 55–65 cm × 4–7(–10) mm, apex acute, base attenuate or rounded, margins scabrous, chartaceous, glabrous on both surfaces. *Synflorescence* 42–90 × 3–6 cm.; synflorescence a spatheate panicle composed of multiple true inflorescences; each true inflorescence consisting of a pair of spike-like racemes subtended by a spatheole. *Racemes* 2, subtended by spatheole 1.7–2.4 cm long, chartaceous, glabrous; lower raceme 2–2.5 cm long; upper raceme 2–2.5 cm long; raceme-based flattened or slightly triangular, hairy; rachis internode slender and flattened, hairy along margins and on the back. *Pedicel of homogamous pair* not swollen. *Peduncle* terete, 0.5–1.3 cm long, glabrous and hairy at the tip. *Spikelet* in pair or triad. *Sessile spikelets* elliptic-lanceolate, 4–5(−5.5) × 0.8–1 mm. *Lower glume* elliptic-lanceolate, 4–5(−5.5) × 0.8–1 mm, apex bifid, narrowly winged or wingless, margins entire, chartaceous, flat or wrinkled on the back, glabrous, 5-nerved. *Upper glume* narrowly boat-shaped, 4–4.5 × 0.4–0.7 mm, apex acuminate, margins entire, chartaceous, 1-keeled with scabrous on keel, glabrous, 1- or 3-nerved. *Florets* 2. *Lower floret* sterile. *Lower lemma* narrowly lanceolate, 3.3–4 × ca. 0.5 mm, apex acute or acuminate, margins ciliate, membranous or hyaline, glabrous, nerveless. *Lower palea* absent. *Upper floret* bisexual. *Upper lemma* 2–3 mm long, apex bifid, awn from sinus; awn geniculate, 0.7–1.1 cm long, scabrous. *Upper palea* absent. *Lodicules* cuneate, ca. 0.4 mm long, truncate. *Stamens*: filament filiform; anther ca. 1.8 mm long. *Pistil*: ovary oblong in outline, ca. 0.8 × 0.2 mm; style 2; stigma plumose. *Caryopsis* not seen. *Pedicelled spikelets* narrowly lanceolate or elliptic, 4–5 × ca. 0.8 mm. *Pedicel* slender and flattened, 2.5–3.3 mm long, hairy along margins and on the back. *Lower glume* narrowly lanceolate or elliptic, 4–5 × ca. 0.8 mm, apex acute, chartaceous, glabrous, 7-nerved. *Upper glume* and *Lower lemma* not observed (Fig. 4 & 5).

**Distribution.** Peninsular Thailand and Peninsular Malaysia.

**Distribution in Thailand.** **Peninsular**: Krabi, Pangnga, Trang.

**Habitat and Ecology.** Open rocky ground and limestone hills or cliffs near sea level.

**Phenology.** Flowering and fruiting from December to April.

**Vernacular name.** **Ya kho daeng** (**
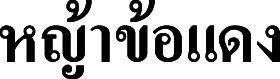
**) (Peninsular).

**Uses.** Not reported.

**Notes.** This species is distinguished by long, slender racemes. All parts are usually glabrous, rarely glabrescent. It occurs on rocky ground, hill slopes, or coastal cliffs.

**Specimens examined.** **Peninsular**: Krabi [Ko Phi Phi, 9 Apr 1930, *A. F. G. Kerr 18895* (BKF, BM, K)]; Pangnga [near Ko Pan Yi, 16 Dec 1918, *Md. Haniff & Nur 4074* (K, SING)]; Trang [Hat Ratchamongkhon, 16 Mar 2018, *P. Wessapak, C. Ngernsaengsaruay, N. Meeprom & W. Boonthasak 454* (BK)].

**Figure 6:**
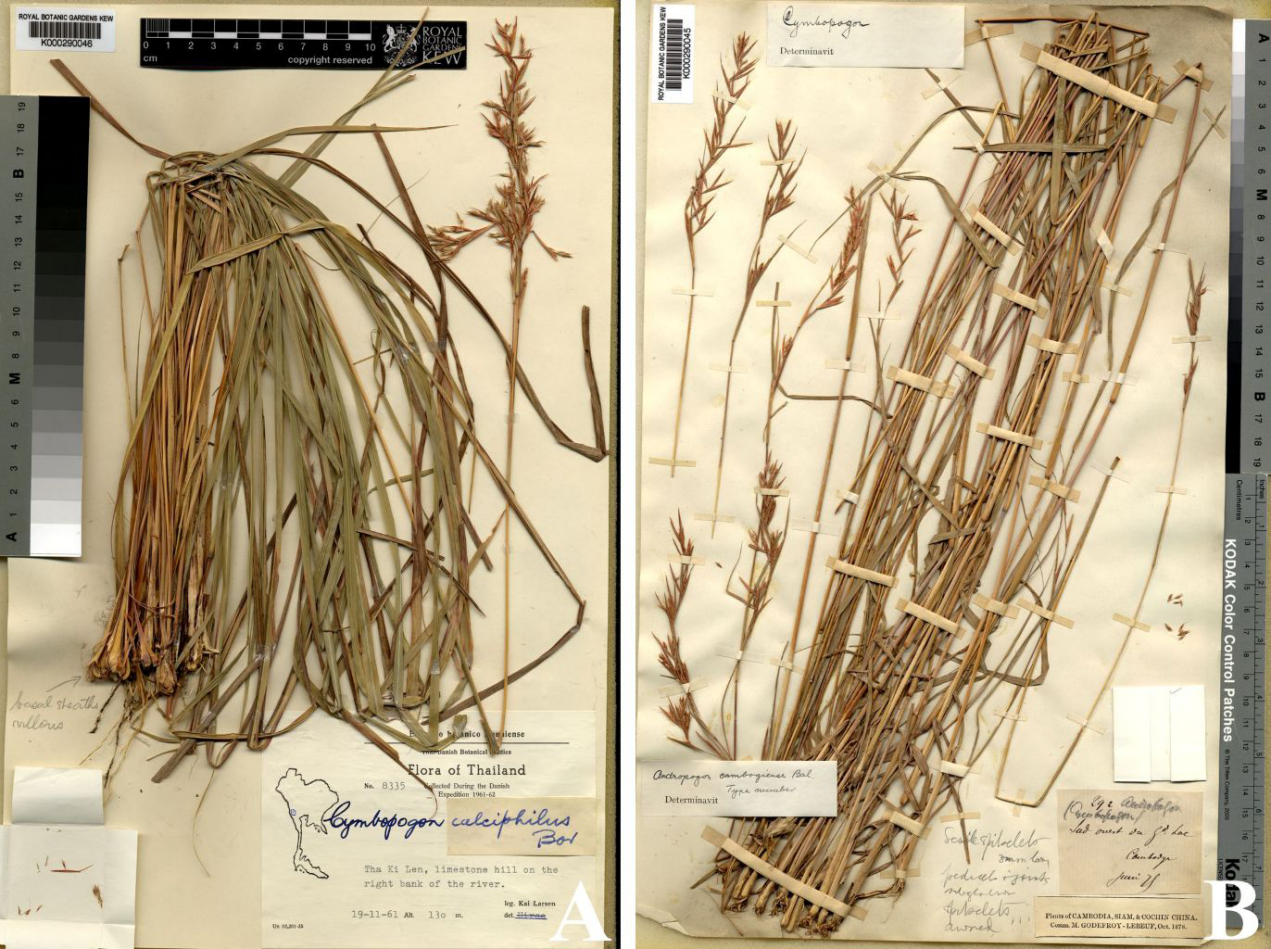
Types of *Cymbopogon*. (A) Holotype of *Cymbopogon calciphilus*, *K. Larsen 8335*, K [K000290046] from Tha Ki Len, Kanchanaburi, Thailand. (B) Lectotype of *Cymbopogon cambogiensis*, *A. Godefroy-Lebeuf 292*, K [K000290045] from “sud-ouest du Grand Lac”, Cambodia, designated here. Photos: ©Board of Trustees of the Royal Botanic Gardens, Kew.

**3. *Cymbopogon calciphilus*** Bor, Dansk Bot. Ark. 23: 157. 1965; Soenarko, Reinwardtia 9: 362. 1977. Type: Thailand, Kanchanaburi, Tha Ki Len, 19 Nov 1961, *K . Larsen 8335* (holotype: **K**! [K000290046] (Fig. 6A); isotype: **A** digital image! [A00023378]).

**Figure 7:**
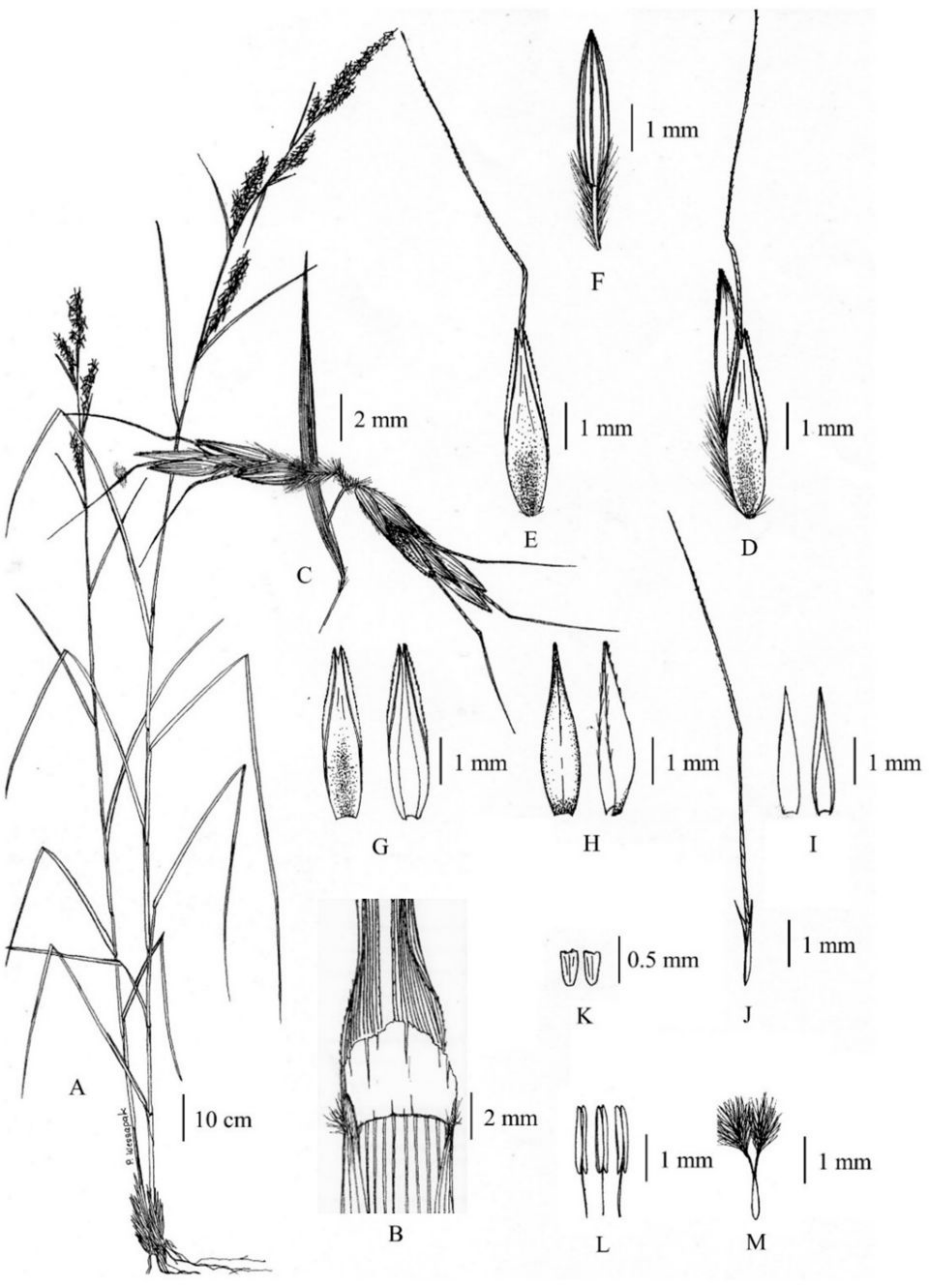
*Cymbopogon calciphilus*. (A) Habit. (B) Ligule. (C) True inflorescence. (D) Paired spikelet (E) Sessile spikelet. (F) Pedicelled spikelets. (G) Lower glumes. (H) Upper glumes. (I) Lower lemmas. (J) Upper lemma with distinct awn. (K) Lodicules. (L) Stamens. (M) Pistil. Photo: Drawn by Paweena Wessapak.

**Figure 8:**
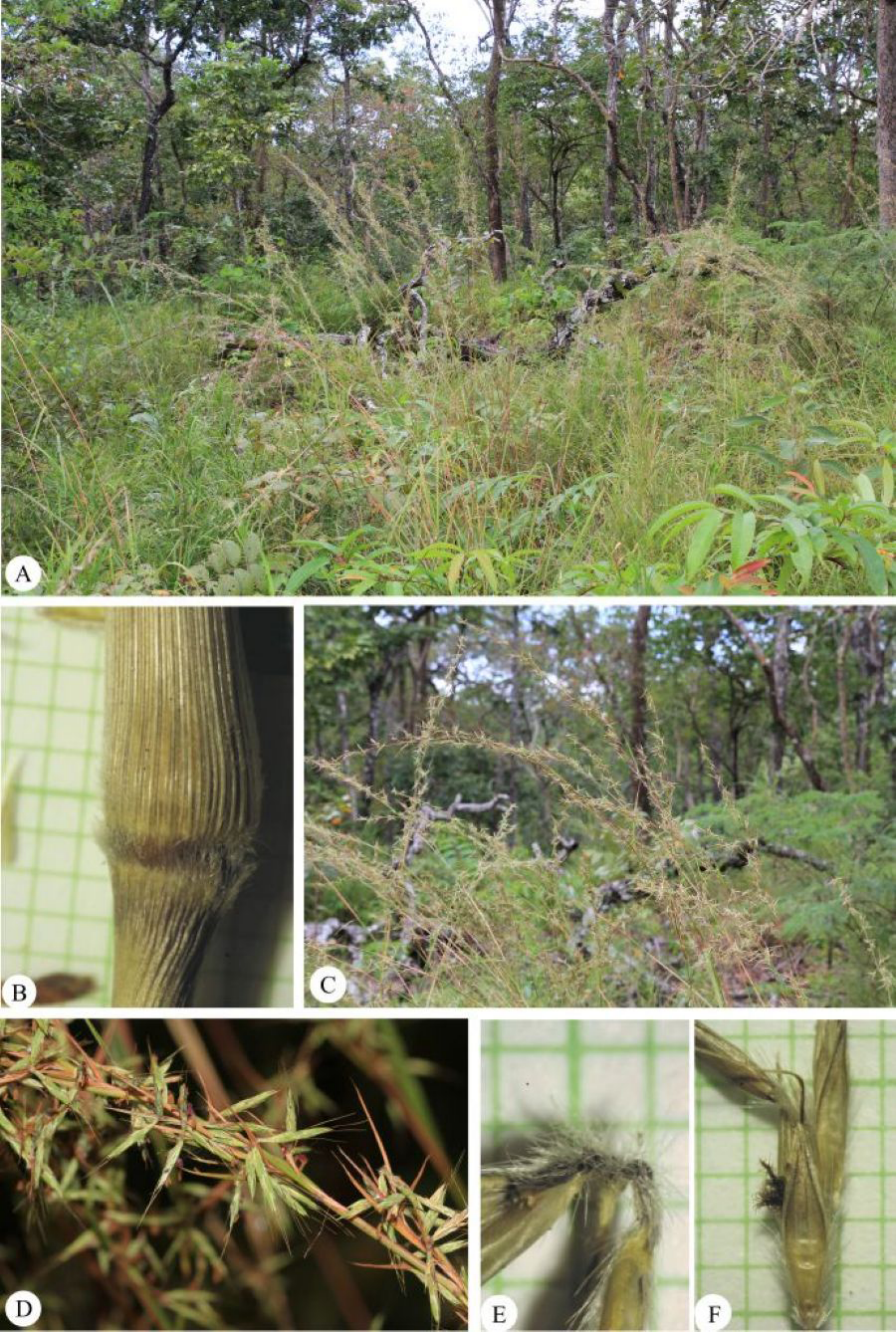
*Cymbopogon calciphilus*. (A) Habitat and habit. (B) Tomentose node. (C) Synflorescences (D) Part of synflorescence. (E) Raceme-based. (F) Spikelets on raceme. Photos: Chatchai Ngernsaengsaruay (A, C–D) and Paweena Wessapak (B, E–F).

Perennial. *Culms* erect, 1–1.5(−1.8) m high (including synflorescences); nodes tomentose, rarely glabrescent; internodes terete, 20–24 cm long, 2–2.5 mm diam., glabrous. *Leaf sheaths* 9–12 cm long, margins entire, base tomentose, rarely glabrescent. *Ligules* chartaceous, 1–1.5 mm long. *Collar* hairy, 1–2 mm long. *Leaf blades* linear, 30–40 cm × 5.5 –7 mm, apex acute, base narrow with hairy triangular patches, margins scabrous, chartaceous, glabrous on both surfaces. *Synflorescence* 60–70 × 6–8 cm.; synflorescence a spatheate panicle composed of multiple true inflorescences; each true inflorescence consisting of a pair of spike-like racemes subtended by a spatheole. *Racemes* 2, subtended by spatheole 1.2–1.6 cm long, chartaceous, glabrous; lower raceme 1.2–1.5 cm long, 4–5 spikelet pairs (including homogamous pair); upper raceme 1.5–1.6 cm long, 4–5 spikelet pairs; raceme-based slightly flattened, hairy; rachis internode flattened, hairy along margins and glabrous on the back. *Pedicel of homogamous spikelet* not swollen. *Peduncle* terete, 5.5–6 mm long, glabrous. *Spikelet* in pair or triad. *Sessile spikelets* elliptic-lanceolate, 4–4.5 × 0.8–1 mm. *Lower glume* elliptic-lanceolate, 4–4.5 × 0.8–1 mm, apex bifid, wingless or narrowly winged, margins entire, usually flat, with or without 1–2-wrinkled on the back, glabrous, usually 2-nerved or nerveless, sometimes obscurely 3(–5)-nerved. *Upper glume* narrowly lanceolate, boat-shaped, ca. 4 × 0.5 mm, apex acuminate, chartaceous, 1-keeled with narrowly winged on keel, glabrous, 3-nerved. *Florets* 2. *Lower floret* sterile. *Lower lemma* narrowly elliptic, 3–3.5 × ca. 0.6 mm, apex acuminate, margins ciliate, hyaline, glabrous, nerveless or obscurely 2-nerved. *Lower palea* absent. *Upper floret* bisexual. *Upper lemma* 1.3–2.3 mm long, apex bifid, awn from sinus; awn geniculate, ca. nine mm long. *Upper palea* absent. *Lodicules* cuneate, ca. 0.3 mm long. *Stamens*: filament filiform; anther brown, ca. 1.8 mm long. *Pistil*: ovary lanceolate in outline; style 2; stigma plumose. *Caryopsis* not seen. *Pedicelled spikelets* elliptic-lanceolate, 3.8–4 × 0.8–1 mm. *Pedicel* 2–3 mm long, hairy on the back and along margins. *Lower glume* elliptic-lanceolate, 3.8–4 × 0.8–1 mm, apex acute, margins hyaline, chartaceous, glabrous, 7–9-nerved (Figs. 7 & 8).

**Distribution.** Endemic to Thailand.

**Distribution in Thailand.** **Northern**: Lampang, Sukhothai; **North-Eastern**: Loei, Sakhon Nakhon, Khon Kaen; **Eastern**: Nakhon Ratchasima, Ubon Ratchathani; **South-Western**: Kanchanaburi; Phetchaburi, Prachuap Khiri Khan.

**Habitat and Ecology.** Limestone hills, at the edges of mixed deciduous and deciduous dipterocarp forests; 50–600 m alt.

**Phenology.** Flowering and fruiting from May to February.

**Vernacular name.** **Ta khrai pa hin pun** (**
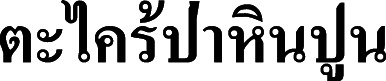
**), proposed here.

**Uses.** Not reported.

**Specimens examined.** **Northern**: Lampang [Hill between Thoen and Li, 600 m alt., 29 Nov 1959, *T. Smitinand & E.C. Abbe 6165* (BKF, K)]; Sukhothai [Locality unspecified, 4 Nov 1971, *J. F. Maxwell 71-693* (AAU)]; **North-Eastern**: Loei [Phu Kradueng, Sam Haek, 400 m alt., 16 Oct 1954, *T. Smitinand 2028* (BKF, K), ibid, 18 Nov 2017, *P. Wessapak 437, 438* (BK)]; Sakhon Nakhon [Phu Phan, 250 m alt., 8 Oct 2017, *P. Wessapak, C. Ngernsaengsaruay, R. Meeboonya, N. Meeprom, W. Boonthasak, T. Ausadamongkol & K. Kampeera 407* (BK)]; Khon Kaen [Phu Wiang, 300 m alt., 5 Feb 1931, *A. F. G. Kerr 19998* (BK, BM, K), ibid., 300 m alt., 28 May 2016, *P. Wessapak, R. Meeboonya & P. Yodboplub 315* (BK), ibid., 29 Oct 2017, *P. Wessapak 422* (BK); **Eastern**: Nakhon Ratchasima [Lat Bua Khao, 7 Nov 1931, *Put 4319* (BK, BM, K)]; Ubon Ratchathani [Phu Chong Na Yoi, 230 m alt., 22 Oct 2017, *P. Wessapak 417* (BK)]; **South-Western**: Kanchanaburi [Erawan National Park, 18 Nov 1970, *M. Lazarides 7424* (C, K); Hin Dat, 50 m alt., 26 Nov 1957, *T. Smitinand 3870* (K); Locality unspecified, 14 Dec 1930, 50 m alt., *A. F. G. Kerr 19876* (BK, BM, K)]; Phetchaburi [Thung Luang, 100 m alt., 8 Nov 1931, *A. F. G. Kerr 20596* (BK, BM, K)]; Prachuap Khiri Khan [Hua Hin, 10 m alt., 6 Nov 1927, *A. F. G. Kerr 13478* (BKF, BM, K); Khao Tao, 20 m alt., 9 Nov 1928, *A. Marcan 2437* (BM, K)].

**4. *Cymbopogon cambogiensis*** (Balansa) E. G. Camus & A. Camus in Lecomte, Fl. Indo-Chine 7: 351. 1922; Soenarko, Reinwardtia 9: 334. 1977.

≡ *Andropogon cambogiensis* Balansa, J. Bot. (Morot) 4: 114. 1890. Type: Cambodia, “sud-ouest du Grand Lac du Cambodge”, 8 Jun 1875, *A. Godefroy-Lebeuf 292* (lectotype, designated here: **K**! [K000290045] (Fig. 6B); isolectotype: **L** digital image! [L0043968]).

= *Cymbopogon siamensis* Bor, Dansk Bot. Ark. 23: 158. 1965. Type: Thailand, “Tapoh”, 27 Dec 1961, *K . Larsen 8989* (holotype: **K**! [K000290044]; isotype: **C**! [C10016854]).

**Figure 9:**
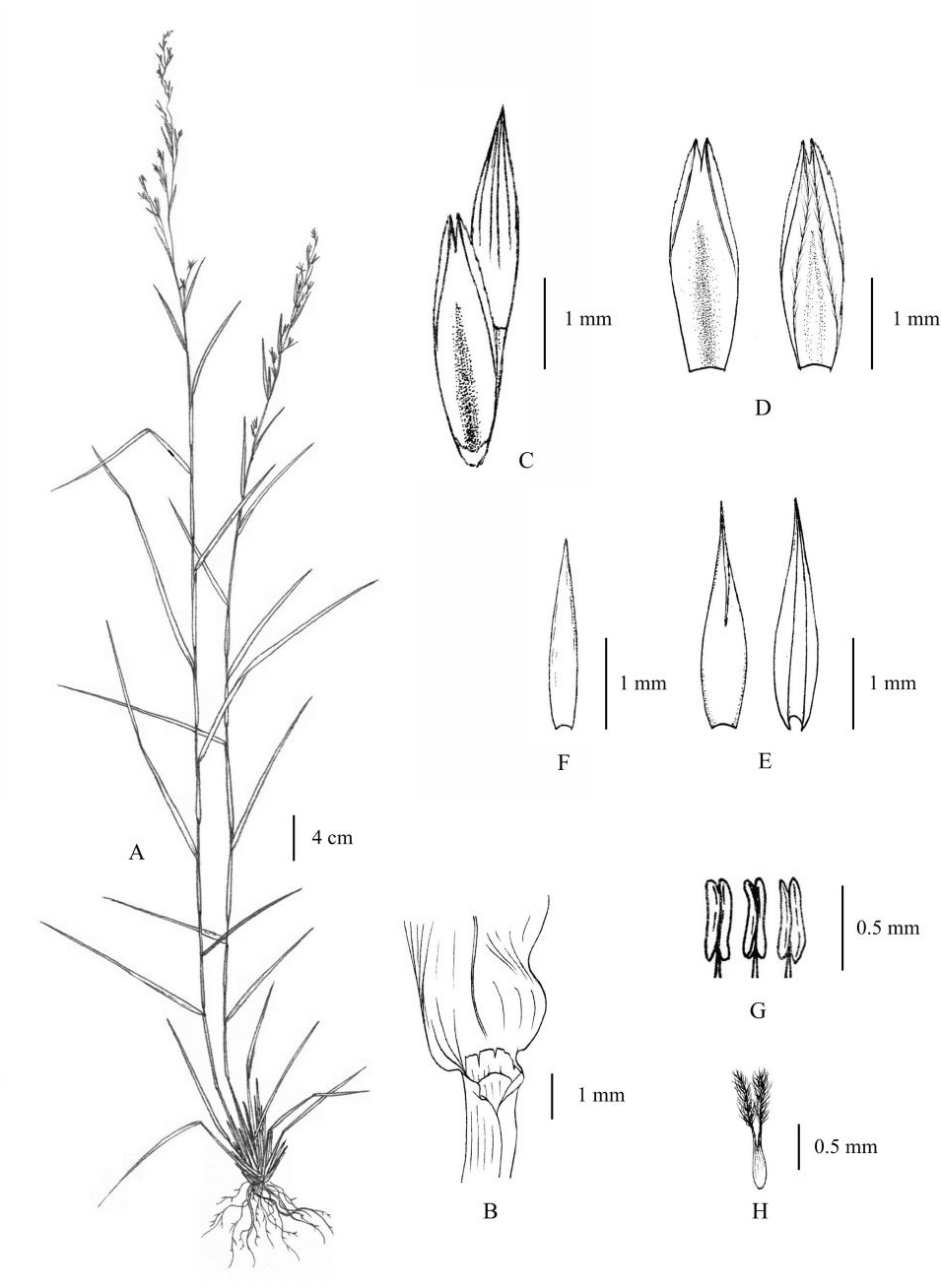
*Cymbopogon cambogiensis*. (A) Habit. (B) Ligule. (C) Paired spikelet. (D) Lower glumes (E) Upper glumes. (F) Lower lemma. (G) Stamens. (H) Pistil. Photo: Drawn by Paweena Wessapak.

Perennial, tufted. *Culms* erect or slightly geniculate, 0.8–1(−1.7) m high (including synflorescences); nodes glabrous; internodes terete, 6–16 cm long, 0.8–2 mm diam., glabrous. *Leaf sheaths* 4–10 mm long, base glabrous, margins entire, glabrous. *Ligules* membranous or chartaceous, 0.5–1.5 mm long. *Collar* glabrous. *Leaf blades* linear, 11–30 × 0.2–1.5 cm, apex acute, base rounded or subcordate, margins scabrous, chartaceous, glabrous on both surfaces. *Synflorescence* 16–50 × 1.5–5 cm.; synflorescence a spatheate panicle composed of multiple true inflorescences; each true inflorescence consisting of a pair of spike-like racemes subtended by a spatheole. *Racemes* 2, subtended by spatheole 1.1–1.8 cm long, chartaceous, glabrous; lower raceme 0.8–1.2 cm long, 4 spikelet pairs (including homogamous pair); upper raceme 1–1.5 cm long, 4 spikelet pairs; raceme-based slightly flattened, usually glabrous or short hairs; rachis internode flattened, hairy along margins. *Pedicel of homogamous pair* not swollen. *Peduncle* terete or subterete, 0.5–2.7 cm long, glabrous. *Spikelet* in pair or triad. *Sessile spikelet* ovate, elliptic or ovate-lanceolate, 2.3–3.2 × 0.7–1 mm. *Lower glume* ovate, elliptic or ovate-lanceolate, 2.3–3.2 × 0.7–1 mm, apex bifid, narrowly or broadly winged, margins entire, a shallow median groove below the middle, chartaceous, glabrous, 2-nerved or nerveless. *Upper glume* lanceolate, boat-shaped, 2–3 × ca. 0.6 mm, apex acute or acuminate, margins entire, chartaceous, 1-keeled, glabrous, nerveless or 1-obscure-nerved. *Florets* 2. *Lower floret* sterile. *Lower lemma* oblong or lanceolate, 2–2.2 × ca. 0.4 mm, apex acute or acuminate, margins entire, hyaline, glabrous, nerveless. *Lower palea* absent. *Upper floret* bisexual. *Upper lemma* 2.2–2.3 mm long, apex acuminate, entire and awnless, or apex bifid, short awn from sinus, 1-nerved. *Upper palea* absent. *Lodicules* cuneate, ca. 0.3 mm long, truncate. Stamens: filament filiform; anther yellow, 0.7–1 mm long. *Pistil*: ovary oblong in outline, ca. 0.3 × 0.1 mm; style 2; stigma plumose. *Caryopsis* not seen. *Pedicelled spikelets* elliptic-lanceolate, 2.8–3.3 × 0.6–1 mm. *Pedicel* slightly flattened, 1–2 mm long, glabrous or short hairs along margins. *Lower glume* elliptic-lanceolate, 2.8–3.3 × 0.6–1 mm, apex acute, margins entire, chartaceous, glabrous, 5- or 7-nerved. *Upper glume* lanceolate, 2.5–3.3 × 0.5–0.8 mm, apex acute, margins entire, chartaceous, 1-nerved. *Lower lemma* lanceolate, 2–3 × ca. 0.5 mm, apex acute, margins ciliate, hyaline, glabrous, nerveless (Fig. 9).

**Distribution.** Vietnam, Cambodia, and Thailand.

**Distribution in Thailand.** **Northern**: Sukhothai, Phitsanulok; **Central**: Chai Nat, Suphan Buri; **South-Eastern**: Sa Kaeo, Chanthaburi; **Peninsular**: Chumphon.

**Habitat and Ecology.** Open scrub, open areas in deciduous forest, and limestone hills; 20–300 m alt.

**Phenology.** Flowering and fruiting from June to January.

**Vernacular name.** **Ya phrik phran** (**
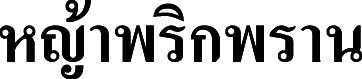
**) (Chai Nat).

**Uses.** Not reported.

**Lectotypification.** *Andropogon cambogiensis* was described by Balansa (1890) from material collected in “sud-ouest du Grand Lac du Cambodge” [south-west of the Great Lake (Tonlé Sap), Cambodia]. The species was later transferred to *Cymbopogon* by Camus & Camus (1922). No type specimen was designated in the protologue, and no herbarium or barcode information was indicated, although a collector number (*Godefroy 292*) was cited. Two specimens referable to the original material, collected by *A. Godefroy-Lebeuf 292* (labelled as *“M. Godefroy-Lebeuf 292*”) from the type locality, have been traced at K [K000290045] and L [L0043968]. As no holotype was indicated, these specimens represent syntypes under Article 9.6 of the International Code of Nomenclature for algae, fungi, and plants (ICN) (Turland et al., 2018). The specimen preserved at K [K000290045], which is more complete and better condition, is designated here as the lectotype; the duplicate at L [L0043968] is designated as the isolectotype, in accordance with Arts. 9.3 and 9.12 of the ICN (Turland et al., 2018).

**Specimens examined.** **Northern**: Sukhothai [Si Satchanalai, 18 Jul 2015, *P. Wessapak, C. Ngernsaengsaruay, S. Chodchoy, N. Meeprom 248* (BK)]; Phitsanulok [Tha Pho, 300 m alt., 27 Dec 1961, *K. Larsen 8989* (C, K)]; **Central**: Chai Nat [Manorom, under 50 m alt., 19 Sep 1930, *A. F. G. Kerr 19677* (BK, BM, K)]; Suphan Buri [Bang Pla Ma, 22 Sep 1930, *A. F. G. Kerr s.n.* (K)]; **South-Eastern**: Sa Kaeo [Aranyaprathet, 50 m alt., 9 Aug 1930, *A. F. G. Kerr 19586* (BK, BM, K)]; Chanthaburi [Makham, 50 m alt., 13 Jun 1963, *K. Larsen 10027* (BKF, C, K)]; **Peninsular**: Chumphon [Ban Pak Khlong, 20 m alt., 12 Jan 1927, *A. F. G. Kerr 11391* (BK, BM, K)].

**5. *Cymbopogon citratus*** (DC.) Stapf, Bull. Misc. Inform. Kew 1906: 322, 357. 1906; Bor, J. Bombay Nat. Hist. Soc. 52: 907. 1953; Bor, Grasses Burma, Ceyl. Ind. & Pakist.: 126. 1960; Gilliland, Re. Fl. Mal. 3: 296. 1971; Soenarko, Reinwardtia 9: 351. 1977.

≡ *Andropogon citratus* DC., Cat. Pl. Horti Monsp. 78. 1813. ‘as *citratum*’. Type: Cult. in Horto Monspeliensis (not traced).

= *Andropogon cerifer* Hack., Fl. Bras. 2, 3: 281. 1883. Type: Brazil, Rio de Janeiro, s.d., *A. F. M. Glaziou 4296* (holotype: **W** digital image! [W1904-0010930]; isotype: **US** digital image! [US00156603 (fragment)]).

Perennial, tufted. *Culms* erect, up to 2 m high (including synflorescences); nodes glabrous; internodes terete, ca. four mm in. *Leaf sheaths* 15–30 cm long, green adaxial side, base glabrous, basal sheath persistent, margins scabrous, glabrous. *Ligules* chartaceous, ca. 1 mm long. *Collar* glabrous. *Leaf blades* linear, 60–90 × 1.5–2 cm, apex acute, base rounded or attenuate, margins scabrous, chartaceous, glabrous on both surfaces. *Synflorescence* 30–50 cm long.; synflorescence a spatheate panicle composed of multiple true inflorescences; each true inflorescence consisting of a pair of spike-like racemes subtended by a spatheole. *Racemes* 2, subtended by reddish spatheole, 1.4–2.5 cm long, chartaceous, glabrous; racemes 1.5–1.7 cm long; rachis internode pilose along margins and on the back. *Pedicel of homogamous pair* not swollen. *Peduncle* glabrous. *Spikelet* in pair or triad. *Sessile spikelets* linear or linear-lanceolate, (5–)6–7.7 × ca. 0.7 mm. *Lower glume* linear or linear-lanceolate, (5–)6–7.7 × ca. 0.7 mm, narrowly winged, chartaceous, flat on the back, slightly concave in the lower part, glabrous, nerveless. *Upper glume* linear or linear-lanceolate, ca. nine mm long, apex acuminate, chartaceous, glabrous. *Upper lemma* narrow, apex entire usually awnless or apex bifid with a mucro ca. 0.2 mm long. *Pedicelled spikelets* linear-lanceolate, 4–7 × 0.7–0.9 mm. *Pedicel* 2.5–4 mm long, hairy. *Lower glume* 4–7 × 0.7–0.9 mm, apex acute, chartaceous, glabrous. *Upper glume* and *Lower lemma* not observed.

**Distribution.** India and Sri Lanka; widely introduced elsewhere for cultivation.

**Distribution in Thailand.** This species is commonly cultivated throughout the country and is known only from cultivation.

**Phenology.** Mostly sterile, rarely flowering or fruiting.

**Vernacular names.** Kha-hom ( 
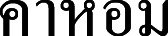
) (Shan-Mae Hong Son); Khrai ( 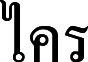) (Peninsular); Cha khrai ( 
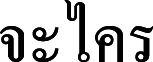
) (Northern); Soet-kroei ( 
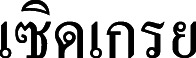
) (Khmer-Surin); **Ta khrai** (**
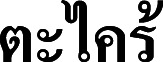
**) (Central); Ho-wo-ta-po ( 
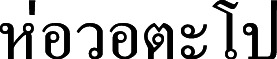
) (Karen-Mae Hong Son); Hua-sing-khai (Khmer-Prachin Buri); Loe-kroei ( 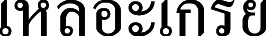) (Khmer-Surin); Fever grass, Lemon grass, Oil grass, West Indian lemon grass (English).

**Uses.** It is widely used in culinary and traditional applications. Its leaves are commonly used to prepare food and beverages, and it is also valued for the extraction of essential oils (our observations). In addition, the essential oils of *C. citratus* are widely applied in traditional medicine, cooking, and tea for flavor, and also have applications in food preservation (Ekpenyong & Akpan, 2017). *Cymbopogon citratus*, or lemongrass, is commonly grown in home gardens throughout Southeast Asia for use as food, beverage flavoring, and a condiment (Oyen & Dung, 1999).

**Notes.** This species has spikelets that are linear to linear-lanceolate. It is readily distinguished by its strong lemon fragrance, which differs from that of other *Cymbopogon* species. Soenarko (1977) noted that the scent of living plants, when the leaf is crushed, is sufficient to recognize *C. citratus*.

Reproductive structures were not observed in this study, as flowers are seldom produced (Chen & Phillips, 2006), and herbarium specimens usually lack inflorescences. In particular, *Cymbopogon* species, including those in this study, are often difficult to find with well-developed inflorescences, as they rarely flower in cultivation, especially for species like *C. citratus*, which is mainly cultivated for its aromatic leaves rather than for reproduction. Some characters included in the description were reported from literature of neighboring countries (Bor, 1953; Bor, 1960; Chen & Phillips, 2006; Veldkamp et al., 2019).

**Specimens examined.** **Peninsular**: Pattani [Nong Chik, 19 Jan 1998, *Y. Sutdhikaran 7* (PSU)].

**6. *Cymbopogon flexuosus*** (Nees ex Steud.) Will. Watson, Gaz. N. W. Ind. 10: 392. 1882; Stapf, Bull. Misc. Inform. Kew 1906: 319, 356. 1906; Bor, J. Bombay Nat. Hist. Soc. 52: 162. 1954; Bor, Grasses Burma, Ceyl. Ind. & Pakist.: 127. 1960; Gilliland, Re. Fl. Mal. 3: 297. 1971; Soenarko, Reinwardtia 9: 353. 1977.

≡ *Andropogon flexuosus* Nees ex Steud., Syn. Pl. Glumac. 1: 388. 1854. Type: India, s.d., *R. Wight 1704* (lectotype, designated by Turner et al. (2019): **E** digital image! [E00174955]; isolectotypes: **K**! [K000245876, K000974914], **LE** [Herb. Trinius 204.1 (fragment), not seen]).

= *Andropogon nardus* L. var. *flexuosus* (Nees ex Steud.) Hack., Monogr. Phan. 6: 603 1889; Hook. f., Fl. Brit. India 7: 207. 1896.

= *Andropogon ampliflorus* Steud., Syn. Pl. Glumac. 1: 388. 1854. Type: Java, s.d., *H. Zollinger 849* (holotype: **P**! [P00740755]; isotypes: **BRI** [BRI-AQ0318680, not seen], **P**! [P00740756]).

= *Cymbopogon travancorensis* Bor, J. Bombay Nat. Hist. Soc. 52: 174. 1954. Type: India, Courtallam, Tennevelly District, 11 Nov 1908, *A. G. Bourne & E. G. Bourne 5309* (holotype: **K** digital image! [K000245877]; isotype: **US** digital image! [US00902245]).

**Figure 10:**
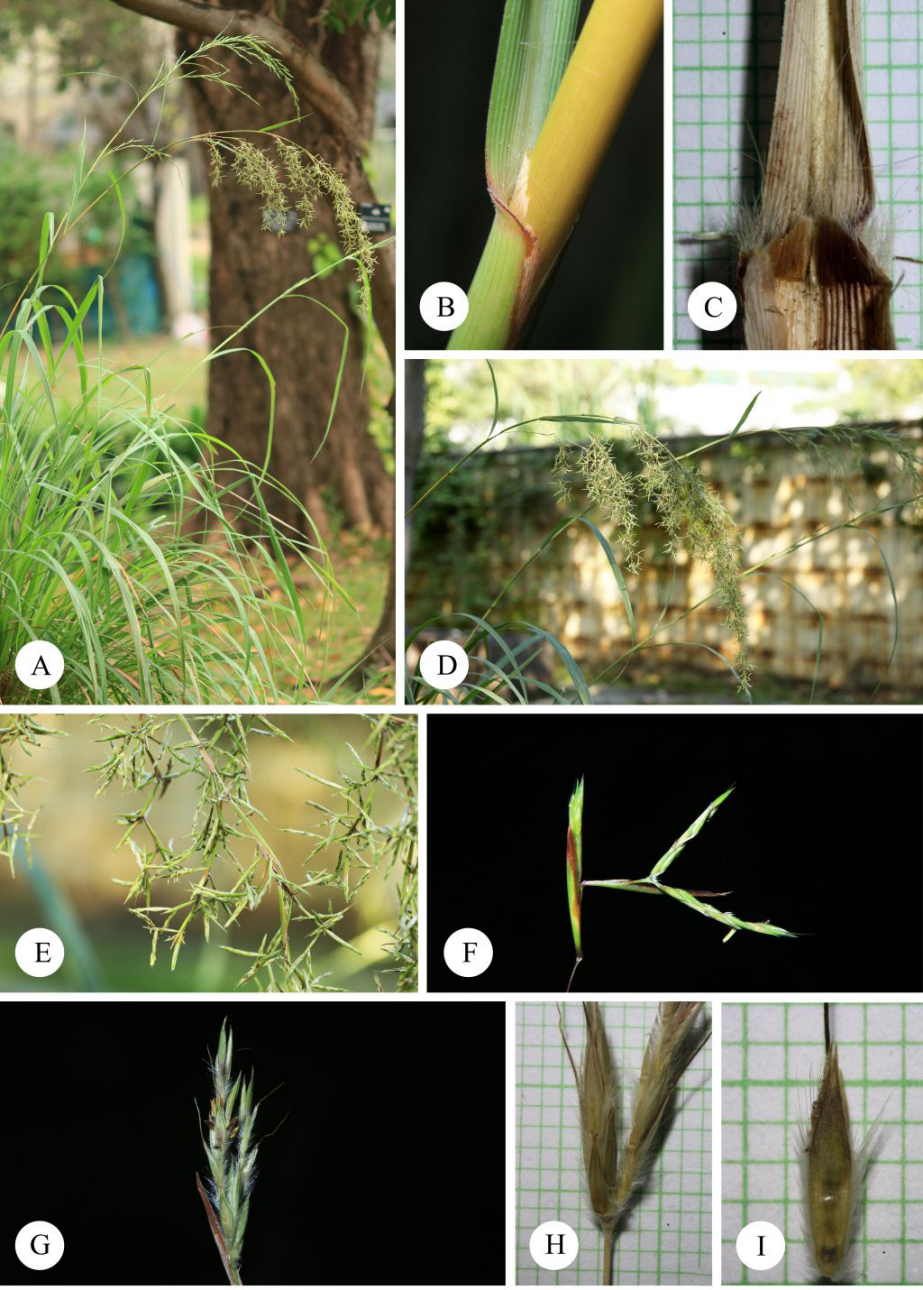
*Cymbopogon flexuosus*. (A) Habit. (B–C) Ligule with long hairs. (D) Synflorescences. (E) Parts of synflorescence. (F) True inflorescence, which in this study refers specifically to a unit consisting of a pair of racemes subtended by a spatheole. (G) Spikelets on raceme. (H) Paired raceme. (I) Sessile spikelet. Photos: Photos: Chatchai Ngernsaengsaruay (A–B, D–G) and Paweena Wessapak (C, H–I).

Perennial, tufted. *Culms* erect, 2–2.5 m high (including synflorescences); nodes bearded; internodes terete or subterete, 27–37 cm long, 2.5–5 mm diam., glabrous. *Leaf sheaths* 13–32 cm long, glabrous, sometimes bearded at the base. *Ligules* chartaceous, 2–3 mm long. *Collar* slightly hairy or glabrous. *Leaf blades* linear 80–130 × 1.4–1.6 cm, apex acute, based rounded, margins scabrous, chartaceous, lower surface glabrous and upper surface glabrous with long hairs present at the base. *Synflorescence* 70–100 × 15–30 cm.; synflorescence a spatheate panicle composed of multiple true inflorescences; each true inflorescence consisting of a pair of spike-like racemes subtended by a spatheole. *Racemes* 2, subtended by spatheole 1.5–2 cm long, chartaceous, glabrous; lower raceme 1.4–1.6 cm long, 3 spikelet pairs (including homogamous pairs); upper raceme 1.4–1.6 cm long, 3 spikelet pairs; raceme-based flattened, hairy; rachis internode flattened, hairy along margins and on the back. *Pedicel of homogamous pair* not swollen. *Peduncle* terete, 0.6–1.5 cm long, pubescent or glabrous with hairy at the tip. *Spikelet* in pair or triad. *Sessile spikelets* narrowly lanceolate, 4–5.8 × 1–1.2 mm. *Lower glume* narrowly lanceolate, 4–5.8 × 1–1.2 mm, apex bifid, narrowly winged, sometimes slightly broadly winged, chartaceous, concave below the middle, sometimes with 2-wrinkled, glabrous, 2-nerved or nerveless, rarely 3-nerved. *Upper glume* narrowly boat-shaped, 4–5.8 × 0.8–1 mm, apex acuminate, margins ciliate, chartaceous, 1-keeled with or without winged on keeled, glabrous, 1-nerved. *Lower floret* sterile. *Lower lemma* lanceolate, 3.8–5 × ca. 0.6 mm, apex acuminate, margins ciliate, membranous or hyaline, glabrous, obscurely 2-nerved or nerveless. *Upper floret* bisexual. *Upper lemma* 2–2.5 mm long, apex bifid with awn from sinus; awn geniculate 1–1.5 cm long. *Stamens*: anther yellow, 1.7–3 mm long. *Pistil*: ovary lanceolate in outline, 0.8–1 × ca. 0.3 mm, style 2; stigma plumose. *Pedicelled spikelets* lanceolate, 4–5 × 0.8–1 mm. *Pedicel* flatten, 2–2.8 mm long, hairy along margins and on the back. *Lower glume* lanceolate, 4–5 × 0.8–1 mm, apex acuminate, margins entire, chartaceous, glabrous, 9-nerved. *Upper glume* lanceolate, 4–4.5 × ca. 0.8 mm, apex acuminate, margins ciliate, chartaceous, glabrous, 3-nerved. *Lower lemma* lanceolate, ca. 4 × 0.6 mm, apex acuminate, margins ciliate, membranous or hyaline, glabrous, nerveless (Fig. 10).

**Distribution.** India, Nepal, Bangladesh, Myanmar, Vietnam, and Thailand.

**Distribution in Thailand.** **Northern**: Chiang Mai; **South-Eastern**: Chon Buri; **Peninsular**: Satun.

**Habitat and Ecology.** Grassland and rocky slope in pine-deciduous dipterocarp forest; 0–1,100 m alt.

**Phenology.** Flowering and fruiting from November to March.

**Vernacular names.** **Ta khrai hom** (**
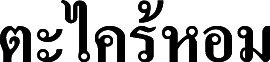
**) (Bangkok); Cochin grass, East Indian lemon grass, Malabar grass (English).

**Uses.** This species is cultivated for the production of aromatic oils. It is the source of valuable aromatic oils, known as “oil of lemon grass” (Bor, 1960). The species is also used as a source of cellulose and for paper production (Oyen & Dung, 1999).

**Note.** This species is characterized by a large spathate panicle with drooping, slender branches and a narrowly winged lower glume of the sessile spikelet. Some specimens from India and Thailand show the lower glume with wings that are clearly broader, as noted by Soenarko (1977) for cultivated plants from Java (Bor, 1960; Chen & Phillips, 2006; Veldkamp et al., 2019). The species can be distinguished by the presence of conspicuous long hairs at the base of the leaf blade. These long hairs are useful for identification but should be considered alongside other characters.

**Specimens examined.** **Northern**: Chiang Mai [Doi Saket, 1,100 m alt., 10 Jan 1969, *T. Smitinand & G. S. 10629* (BKF)]; **Central**: Bangkok [Cultivated, Kasetsart University, 13 Feb 2017, *P. Wessapak 353* (BK)]; **South-Eastern**: Chon Buri [Cultivated, Si Racha, 31 Mar 1923, *A. F. G. Kerr 6956* (BK, BM, K); Si Racha, 24 m alt., 22 Nov 1927, *D. J. Collins 1892* (BK, K)]; **Peninsular**: Satun [Ko Batuang, 50 m alt., 13 Jan 1928, *A. F. G. Kerr 14057* (BK, BM, K)].

**7. *Cymbopogon khasianus*** (Hack.) Stapf ex Bor, Indian Forest Rec., Bot. 1: 92. 1938; Bor, Grasses Burma, Ceyl. Ind. & Pakist.: 128. 1960; Soenarko, Reinwardtia 9: 346. 1977.

**Figure 11:**
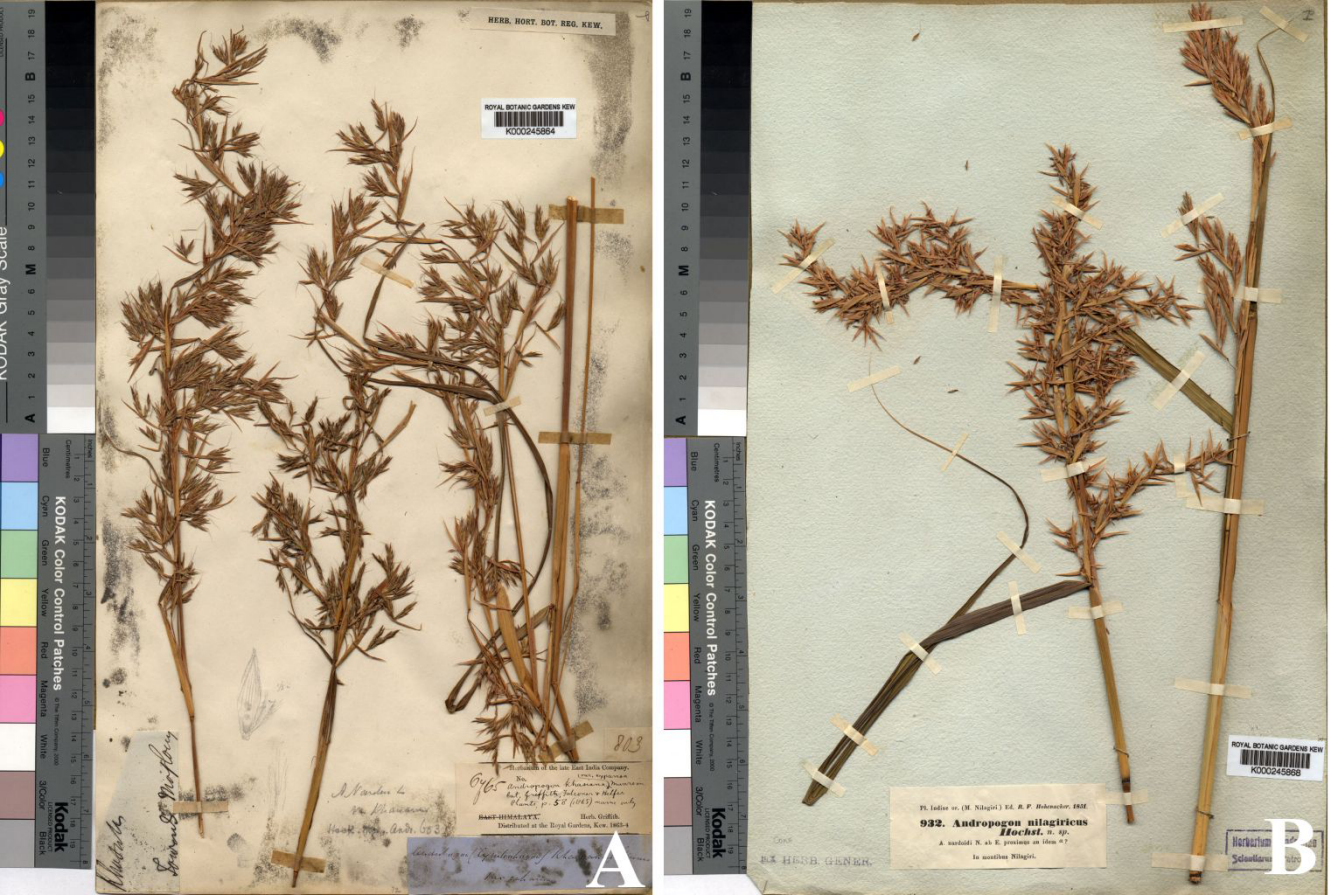
Types of *Cymbopogon*. (A) Lectotype of *Cymbopogon khasianus*, *W. Griffith 6765*, K [K000245864] from Khasia Hills, India. (B) Lectotype of *Cymbopogon nardus* var. *confertiflorus*, a new synonym of * Cymbopogon nardus*, *R. F. Hohenacker 932*, K [K000245868] from Nilagiri Hills, India, designated here. Photos: ©Board of Trustees of the Royal Botanic Gardens, Kew.

≡ *Andropogon nardus* L. var. *khasianus* Hack. in A. DC. & C. DC., Monogr. Phan. 6: 603. 1889; Hook. f., Fl. Brit. India 7: 206. 1896. Type: India, Khasia Hills, s.d., *W. Griffith 6765* (lectotype, designated here: **K**! [K000245864] (Fig. 11A); isolectotype: **W** digital image! [W0027085]).

**Figure 12:**
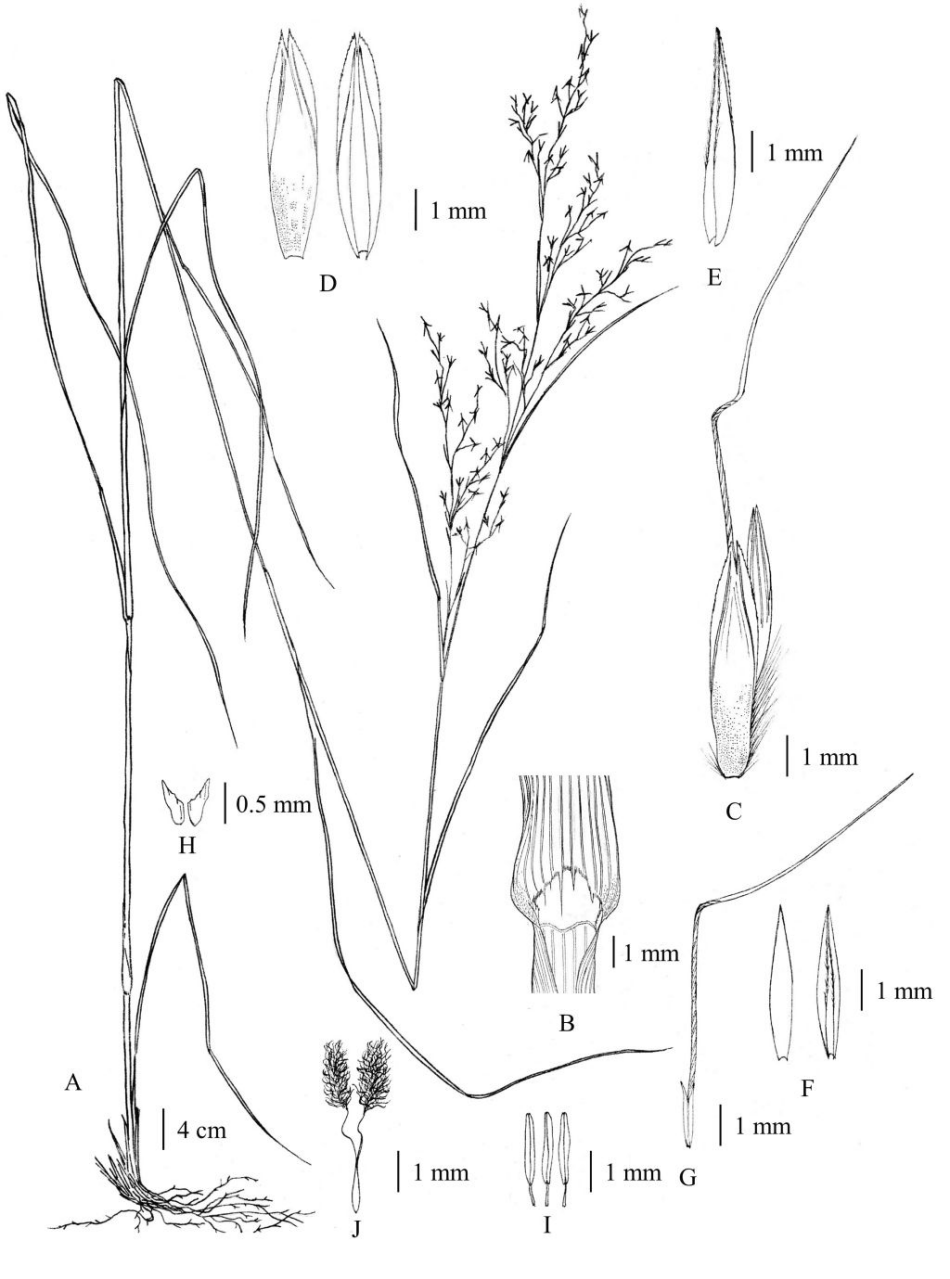
*Cymbopogon khasianus*. (A) Habit. (B) Ligule. (C) Paired spikelet. (D) Lower glumes. (E) Upper glume. (F) Lower lemmas. (G) Upper lemma with distinct awn. (H) Lodicules. (I) Stamens. (J) Pistil. Photo: Drawn by Paweena Wessapak.

**Figure 13:**
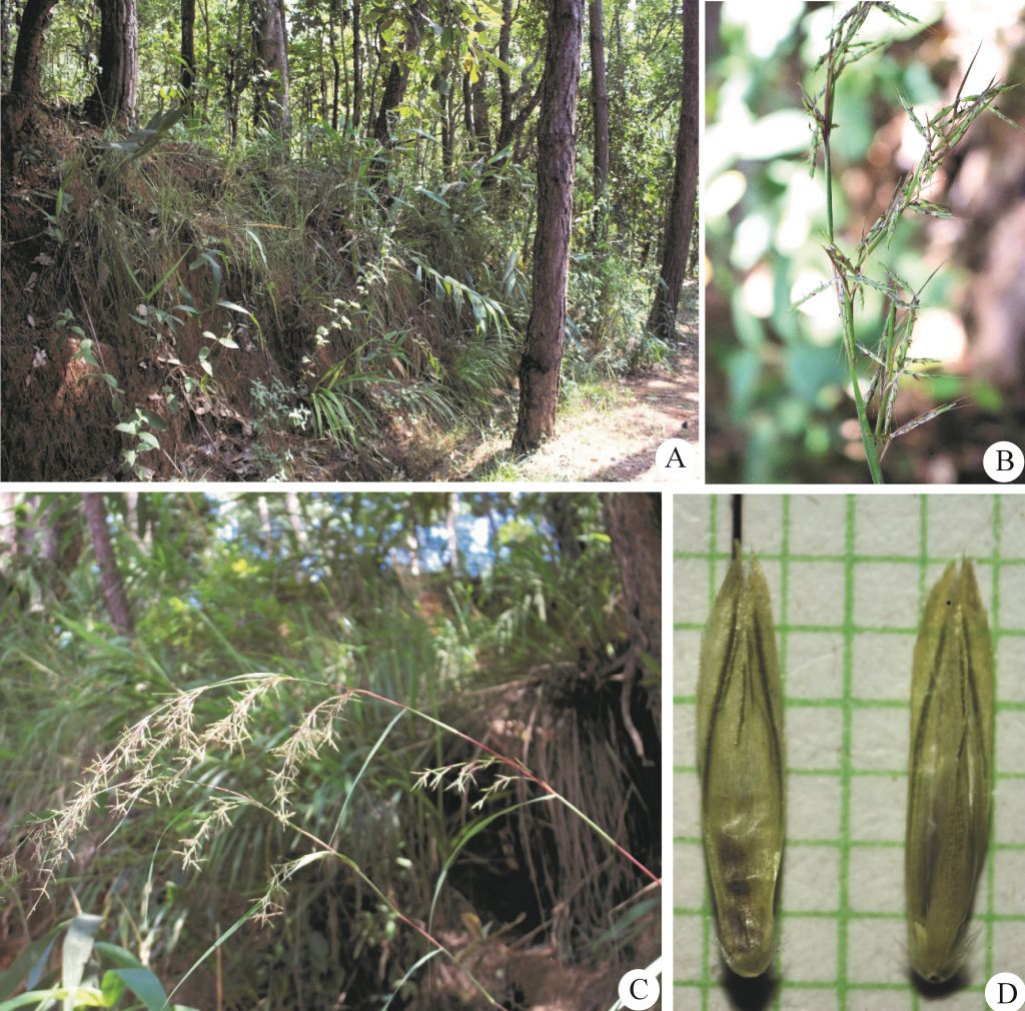
*Cymbopogon khasianus*. (A) Habitat and habit. (B) Part of synflorescence. (C) Synflorescences. (D) Sessile spikelets in the front (left) and in the back (right). Photos: Paweena Wessapak.

Perennial. *Culms* 2–2.5 m high (including synflorescences); nodes glabrous or beard; internodes 23–50 cm long, 3–7 mm diam., glabrous. *Leaf sheaths* 10–50 cm long, glabrous. *Ligules* membranous or chartaceous, 3–4 mm long. *Collar* hairy. *Leaf blades* linear 0.5–1.3 m × 0.8–1.3 cm, apex acute, base usually narrow or rounded, margins scabrous, chartaceous, glabrous on both surfaces. *Synflorescence* 36–100 × 6–15 cm.; synflorescence a spatheate panicle composed of multiple true inflorescences; each true inflorescence consisting of a pair of spike-like racemes subtended by a spatheole. *Racemes* 2, subtended by spatheole 1.5–3.2 cm long, chartaceous, glabrous; lower raceme 1.5–2.4 cm long, 3–6 spikelet pairs (including homogamous pair); upper raceme 1.7–2.6 cm long, 3–7 spikelet pairs; raceme-based flattened, hairy; rachis internode flattened, hairy along margins, glabrous on one side. *Pedicel of homogamous pair* not swollen. *Peduncle* 0.8–1.4 cm long, glabrous. *Spikelet* in pair or triad. *Sessile spikelets* oblong-lanceolate, usually narrow at base, 5.2–6 × ca. 1 mm. *Lower glume* oblong-lanceolate, 5.2–6 × ca. 1 mm, apex bifid, broadly winged, chartaceous, concave below the middle, usually 1- or 3-wrinkled, glabrous, 4–5-nerved. *Upper glume* boat-shaped, 4.8–5.2 × ca. 0.8 mm, apex acuminate, margins ciliate, chartaceous, 1-keeled, glabrous, 3-nerved. *Florets* 2. *Lower floret* sterile. *Lower lemma* oblong or narrowly lanceolate, 3.8–4 × ca. 0.4 mm, apex acute, margins ciliate, hyaline, glabrous, nerveless. *Lower palea* absent. *Upper floret* bisexual. *Upper lemma* ca. 2.5 mm long, apex bifid, awn from sinus; awn geniculate, 1.2–1.4 cm long. *Upper palea* absent. *Lodicules* ca. 0.5 mm long, acute. *Stamens*: anther yellow, 1.6–1.8 mm long. *Pistil*: ovary elliptic in outline, ca. 1 mm long; style 2; stigma plumose. *Pedicelled spikelets* lanceolate, 5–5.4 × ca. 1 mm. *Pedicel* flattened, ca. 2.5 mm long, hairy along margins, glabrous on the back. *Lower glume* lanceolate, 5–5.4 × ca. 1 mm, apex acuminate, chartaceous, glabrous, 10-nerved. *Upper glume* lanceolate, 4.3–4.5 × ca. 1 mm, acuminate, margins ciliate, chartaceous, glabrous, 3-nerved. *Lower lemma* lanceolate-oblong, ca. 4 × 0.7 mm, acuminate, margins ciliate, membranous, glabrous, nerveless (Figs. 12 & 13).

**Distribution.** India (Assam), Bhutan, Bangladesh, Myanmar, China (Yunnan, Guangxi), and Thailand.

**Distribution in Thailand.** **Northern**: Chiang Mai.

**Habitat and Ecology.** By roadsides, in open grassy forest, at the edge of deciduous forest, and at the edge of lower montane forest 400–1,170 m alt.

**Phenology.** Flowering and fruiting in November.

**Vernacular name.** **Ta khrai pa cho dok khaep** (**
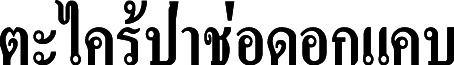
**), proposed here.

**Uses.** Not reported.

**Lectotypification.** *Andropogon nardus* var. *khasianus* was described by Hackel (1889) based on three gatherings from eastern India, near Sylhet (in the protologue “India or. pr. Silhet”): *Wall. 8794H*, *Griffith 6764*, and *Griffith 6765*. Examination of the specimen *N. Wallich Cat. 8794H*, collected by W. Gomez from Sylhet (in the protologue “Sillet”) and preserved at K [K001128222], showed that it represents *Andropogon schoenanthus* L. (= *Cymbopogon schoenanthus* (L.) Spreng.) rather than *A. nardus* var. *khasianus*.

The taxon was later raised to species rank as *Cymbopogon khasianus* by Bor (1938). As Hackel did not select a type specimen or cite the depository of the original material, all specimens associated with these gatherings constitute original material. Material corresponding to *W. Griffith 6764* from the Khasia Hills is represented by a single specimen at K [K000245863], whereas *W. Griffith 6765* from the same locality is represented by two specimens at K [K000245864] and W [W0027085]. These specimens are therefore regarded as syntypes in accordance with Art. 9.6 of the ICN (Turland et al., 2018). Among them, the specimen *W. Griffith 6765* at K [K000245864] is designated here as the lectotype, and the corresponding specimen at W [W0027085] is designated as the isolectotype, in accordance with Arts. 9.3 and 9.12 of the ICN (Turland et al., 2018). The specimen *W. Griffith 6765* at K [K000245864] was selected due to its superior preservation and completeness, representing the best morphological characteristics of the species.

**Specimens examined.** **Northern**: Chiang Mai [Doi Suthep, 762 m alt., 12 Nov 1911, *A. F. G. Kerr 1554B* (BM, K); Mae Rim, 400 m alt., 23 Nov 1995, *W. Nanakorn et al. 5327* (QBG); Tha Pha, Mae Chaem, 1,170 m alt, 25 Nov 2017, *P. Wessapak 443, 444* (BK)].

**8. *Cymbopogon martini*** (Roxb.) Will. Watson, Gaz. N. W. Ind.: 392. 1882; Stapf, Bull. Misc. Inform. Kew 1906: 318, 359. 1906; Bor, Grasses Burma, Ceyl. Ind. & Pakist.: 129. 1960; Gilliland, Re. Fl. Mal. 3: 295. 1971; Soenarko, Reinwardtia 9: 330. 1977. ‘as *martinii*’.

≡ *Andropogon martini* Roxb., Fl. Ind. 1: 280. 1820. Type: India, Calcutta, Calcutta Botanic Garden, 18 Feb 1798, *T. Hardwicke s.n.* (neotype, designated by Turner et al. (2019): **BM** digital image! [BM000959800]).

Perennial, tufted. *Culms* erect, 1.5–2 m high (including synflorescences); nodes glabrous; internodes terete, 5–17 cm long, 2–3 mm diam., glabrous. *Leaf sheaths* 7–12 cm long, margins entire, glabrous. *Ligules* chartaceous, 1–1.5 mm long. *Collars* glabrous. *Leaf blades* linear, 35–50 × 0.8–1.5 cm, apex acute, base cordate, often amplexicaul, tomentose, chartaceous, glabrous on both surfaces. *Synflorescence* ca. 60 × four cm.; synflorescence a spatheate panicle composed of multiple true inflorescences; each true inflorescence consisting of a pair of spike-like racemes subtended by a spatheole. *Racemes* 2, subtended by spatheole 2.4–3 cm long, chartaceous, glabrous; lower raceme 1.5–2 cm long; upper raceme 1.5–2 cm long; raceme-based flattened, broad, hairy; rachis internode flattened, hairy along margins, glabrous or hairy on the back. *Pedicel of homogamous pair* swollen. *Peduncle* subterete or angular, 1.7–2 cm long, glabrous and pubescent at the tip. *Spikelet* in pair or triad. *Sessile spikelets* lanceolate, 4.2–4.5 × ca. 1 mm. *Lower glume* lanceolate, 4.2–4.5 × ca. 1 mm, apex bifid, narrowly or broadly winged, margins entire, chartaceous, a deep median groove below the middle, glabrous, 2- or 4-nerved. *Upper glume* lanceolate, boat-shaped, 4.2–4.5 × ca. 0.8 mm, chartaceous, 1-keeled with winged on keel, glabrous, 1-nerved. *Florets* 2. *Lower floret* sterile. *Lower lemma* lanceolate, 2.8–3 × ca. 0.6 mm, apex acute, margins ciliate, hyaline, glabrous, nerveless. *Lower palea* absent. *Upper floret* bisexual. *Upper lemma* two mm long, apex bifid, awn from sinus; awn geniculate, 1.4–1.5 cm long, scabrous. *Upper palea* absent. *Lodicules* cuneate, ca. 0.5 mm long. *Stamens* not seen. *Pistil*: ovary oblong in outline, 0.3–1 × ca. 0.2 mm; style 2; stigma plumose. *Caryopsis* not seen. *Pedicelled spikelet* lanceolate, 4–4.2 × ca. 1 mm. *Pedicel* flattened, 2–2.5 mm long, hairy along margins, glabrous or hairy on the back. *Lower glume* lanceolate, 4–4.2 × ca. 1 mm, apex acute, margins entire, subchartaceous, glabrous, 10-nerved. *Upper glume* lanceolate 4–4.2 × 0.8–1 mm, apex acuminate, margins ciliate, chartaceous, slightly 1-keeled, glabrous 1-nerved. *Lower lemma* oblong, 3.5–3.8 × ca. 0.5 mm, apex acute, margins ciliate, hyaline, glabrous, nerveless.

**Distribution.** Pakistan, India, Nepal, Bangladesh, Myanmar, Vietnam, and Thailand.

**Distribution in Thailand.** **Northern**: Chiang Mai; **North-Eastern**: Phetchabun; **South-Western**: Kanchanaburi; **South-Eastern**: Chon Buri.

**Habitat and Ecology.** Open areas in deciduous forest and along roadbanks, common on limestone or granite hills; 100–625 m alt.

**Phenology.** Flowering and fruiting from October to January.

**Vernacular names.** **Ta khrai daeng** (**
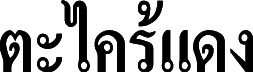
**) (Central); ta khrai hom ( 
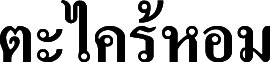
) (Song Khla); Geranium grass, Ginger grass, Indian geranium, Martin’s citronella grass, Martin’s lemon grass, Palmarosa, Palmarosa grass, Rosha grass (English).

**Uses.** This species is cultivated in tropical regions as a source of aromatic raw materials. Its essential oils are commonly known as palmarosa oil and ginger grass oil (Bor, 1960).

**Notes.** The diagnostic characters of *Cymbopogon martini* are a broadly winged lower glume of the sessile spikelet with a deep median groove and a leaf blade base that is distinctly cordate and often amplexicaul. The latter character is particularly useful for distinguishing *C. martini* from *C. annamensis*. In addition, *C. martini* generally has larger culms and broader leaf blades than *C. annamensis*. Material from cultivated areas usually exhibits robust culms and broad leaf blades (up to 2.5 mm wide), whereas material from natural habitats tend to have more slender culms and narrower leaf blades; these vegetative characters sometimes overlap with those of *C. annamensis*.

**Specimens examined.** **Northern**: Chiang Mai [300 m alt., 3 Nov 1960, *Anonymous s.n.* (BK33204)]; **North-Eastern**: Phetchabun [At km 25 on road 12 Lom Sak-Khon Kaen, 625 m alt., 25 Oct 2001, *S. Laegaard & M. Norsaengsri 21786* (AAU, QBG)]; **South-Western**: Kanchanaburi [Si Sawat, 100 m alt., 11 Jan 1926, *A. F. G. Kerr 10192* (BM, BK, K); Khao Salop National Park, 18 Nov 1970, *M. Lazarides 7422* (K)]; **South-Eastern**: Chon Buri [Locality unspecified, 24 Nov 1970, *M. Lazarides 7448* (K)].

**9. *Cymbopogon microstachys*** (Hook. f.) Soenarko, Reinwardtia 9: 364. 1977.

≡ *Andropogon nardus* L. var. *microstachys* Hook. f., Fl. Brit. India 7: 207. 1896. Type: India, Dec 1886, *G. Mann 5* (lectotype, designated by Soenarko (1977): **K**! [K000245880]).

= *Cymbopogon flexuosus* (Nees ex Steud.) Will. Watson var. *microstachys* (Hook. f.) Bor, J. Bombay Nat. Hist. Soc. 52: 162. 1954; Bor, Grasses Burma, Ceyl. Ind. & Pakist.: 127. 1960.

**Figure 14:**
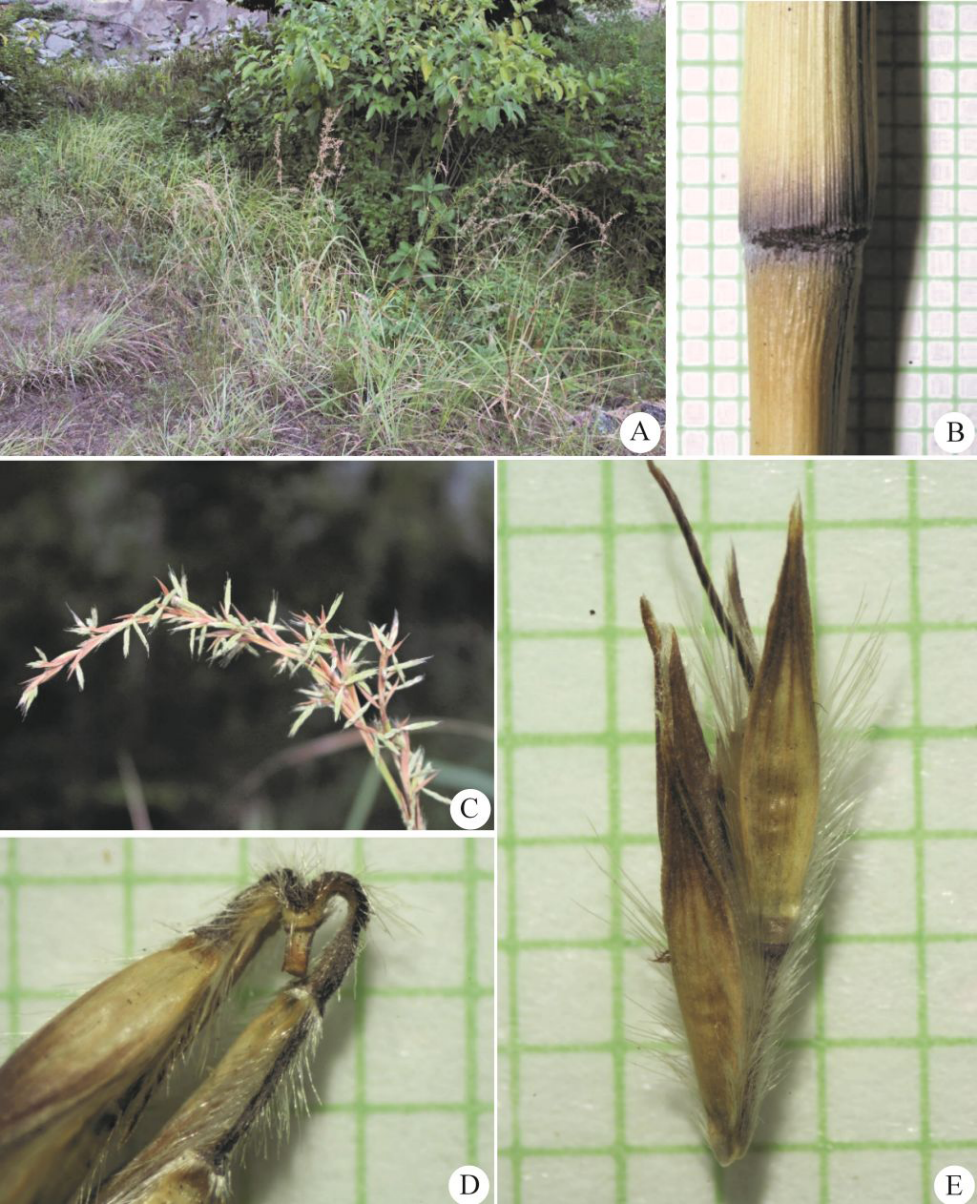
*Cymbopogon microstachys*. (A) Habitat and habit. (B) Node. (C) Synflorescence. (D) Raceme-based. (E) Paired spikelet. Photos: Paweena Wessapak.

Perennial, tufted. *Culms* 1.3–3 m high (including synflorescences); nodes glabrous; internodes terete, 16–50 cm long, 1.5–7 mm diam. glabrous. *Leaf sheaths* 8–38 cm long, glabrous, sometime glabrescent at base. *Ligules* chartaceous, 3.5–5.5 mm long. *Collar* glabrous or tomentose. *Leaf blades* linear, 50–100 × 1–1.4 cm, apex acute, base attenuate or rounded, sometimes tomentose, margins scabrous, chartaceous, glabrous on both surfaces. *Synflorescence* 40–150 × 3–9 cm.; synflorescence a spatheate panicle composed of multiple true inflorescences; each true inflorescence consisting of a pair of spike-like racemes subtended by a spatheole. *Raceme* 2, nearly deflexed at maturity, subtended by spatheole 0.9–2.2 cm long, chartaceous, glabrous; lower raceme 0.9–1.5 cm long, 3–5 spikelet pairs (including homogamous pair); upper raceme 1–1.5 cm long, 3–5 spikelet pairs; raceme-based slightly flattened, hairy; rachis internode flattened, hairy along margins and on the back. *Pedicel of homogamous pair* not swollen. *Peduncle* terete, 4–8 mm long, glabrous. *Spikelet* in pair or triad. *Sessile spikelets* lanceolate, 3.3–4.3 × 0.7–1 mm. *Lower glume* lanceolate, 3.3–4.3 × 0.7–1 mm, apex bifid, narrowly winged or wingless, chartaceous, flat or 1–2-wrinkled on the back, glabrous, (2–)3–4(–5)-nerved. *Upper glume* lanceolate, boat-shaped, 3–4.3 × ca. 0.7 mm, apex acuminate, margins ciliate, chartaceous, 1-keeled with winged on keel, glabrous, 1-nerved. *Florets* 2. *Lower floret* sterile. *Lower lemma* lanceolate 2.8–4 × ca. 0.5 mm, apex acute, margins ciliate, membranous or hyaline, glabrous, nerveless. *Lower palea* absent. *Upper floret* bisexual. *Upper lemma* 2–2.8 mm long, apex bifid, awn from sinus; awn geniculate, 0.7–1 cm long. *Upper palea* absent. *Lodicules* cuneate, ca. 0.5 mm long, truncate. *Stamens*: filament filiform; anther yellow, 1.3–1.8 mm long. *Pistil*: ovary narrowly elliptic in outline, 0.2–0.7 × ca. 0.2 mm; style 2; stigma plumose. *Caryopsis* not seen. *Pedicelled spikelets* lanceolate, 2.7–3.6 × 0.5–0.8 mm. *Pedicel* flattened, 1.8–2 mm long, hairy along margins and on the back. *Lower glume* lanceolate, 2.7–3.6 × 0.5–0.8 mm, apex acuminate, subchartaceous or membranous, with or without 1-keeled, glabrous, 9–11-nerved. *Upper glume* lanceolate, 2.4–3.4 × ca. 0.6 mm, apex acute, margins ciliate, membranous or chartaceous, glabrous, 3-nerved. *Lower lemma* lanceolate, 2.4–3.2 × 0.3–0.7 mm, apex acuminate, margins ciliate, membranous or hyaline, glabrous, nerveless (Fig. 14).

**Distribution.** India, Myanmar, China (Yunnan), and Thailand.

**Distribution in Thailand.** **North-Eastern**: Phetchabun; **Central**: Lop Buri; **South-Western**: Kanchanaburi; **Peninsular**: Surat Thani, Krabi.

**Habitat and Ecology.** Open rocky grounds along roadsides, limestone hills, hillsides, and the edge of deciduous forest; 100–870 m alt.

**Phenology.** Flowering and fruiting from September to March.

**Vernacular name.** **Ta khrai pa cho kha naeng san** (**
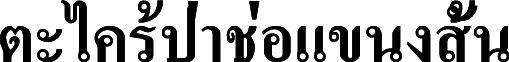
**), proposed here.

**Uses.** The chemical composition of the essential oil of this species has been studied (Mathela, 1989; Rout, Sahoo & Rao, 2005), but its utilization has not been reported.

**Notes.** This species resembles *Cymbopogon calciphilus*, but its basal sheaths and triangular patches are usually glabrous. The diagnostic characters of *C. microstachys* include short racemes and small spikelets. Deflexed racemes are usually present at maturity.

**Specimens examined. North-Eastern**: Phetchabun [Nam Nao, 870 m alt., 30 Sep 2017, *P. Wessapak 403* (BK)]; **Central**: Lop Buri [Chai Badan, 100 m alt., 16 Dec 1923, *A. F. G. Kerr 8013* (BK, BM, K)]; **South-Western**: Kanchanaburi [Kaeng Pralom, 400 m alt., 25 Dec 1961. *K. Larsen 8939* (C, K)]; **Peninsular**: Surat Thani [Ko Phangan, 300 m alt., 23 Sep 1928, *A. F. G. Kerr 16074* (BK, BM, K); ibid., 17 Sep 2016, *P. Wessapak, C. Ngernsaengsaruay, R. Meeboonya & N. Meeprom 333, 334, 335* (BK)]; Krabi [Ko Lanta Yai, 12 Nov 1966, 20 m alt., *B. Hansen & T. Smitinanad 12252* (BKF, C, K, SING); ibid., 16 Mar 2018, *P. Wessapak, C. Ngernsaengsaruay, N. Meeprom & W. Boonthasak 449, 450, 452, 453* (BK)].

**10. *Cymbopogon nardus*** (L.) Randle, Cat. Afr. Pl. 2: 155. 1899; Bor, J. Bombay Nat. Hits. Soc. 51: 903. 1953; Bor, Grasses Burma, Ceyl. Ind. & Pakist.: 130. 1960; Soenarko, Reinwardtia 9: 349. 1977.

≡ *Andropogon nardus* L., Sp. Pl. 2: 1046. 1753. Type: Herb. Hermann 2: 66, No.45 (lectotype, designated by *Clayton & Renvoize (1982)*: **BM** digital image! [BM000594628]).

= *Andropogon nilagiricus* Hochst. in Exsicc. (Pl. Hohenack.) 1851: n.^∘^932. 1851, *nom. superfl.*

= *Cymbopogon nardus* (L.) Randle var. *confertiflorus* (Steud.) Bor, J. Bombay Nat. Hits. Soc. 51: 905. 1953; Bor, Grasses Burma, Ceyl. Ind. & Pakist.: 130. 1960; Soenarko, Reinwardtia 9: 350. 1977.

= *Cymbopogon confertiflorus* (Steud.) Stapf, Bull. Misc. Inform. Kew 1906: 318, 355. 1906.

= *Andropogon confertiflorus* Steud., Syn. Pl. Glumac. 1: 385. 1854. Type: India, Nilagiri Hills, 1851, *R. F. Hohenacker 932* (lectotype, designated here: **K**! [K000245868] (Fig. 11B); isolectotypes: **BM**! [BM00959804], **P**! [P00745754], **E** digital image! [E00393597], **FI** digital image! [FI012282], **S** digital image! [S-G-402]).

**Figure 15:**
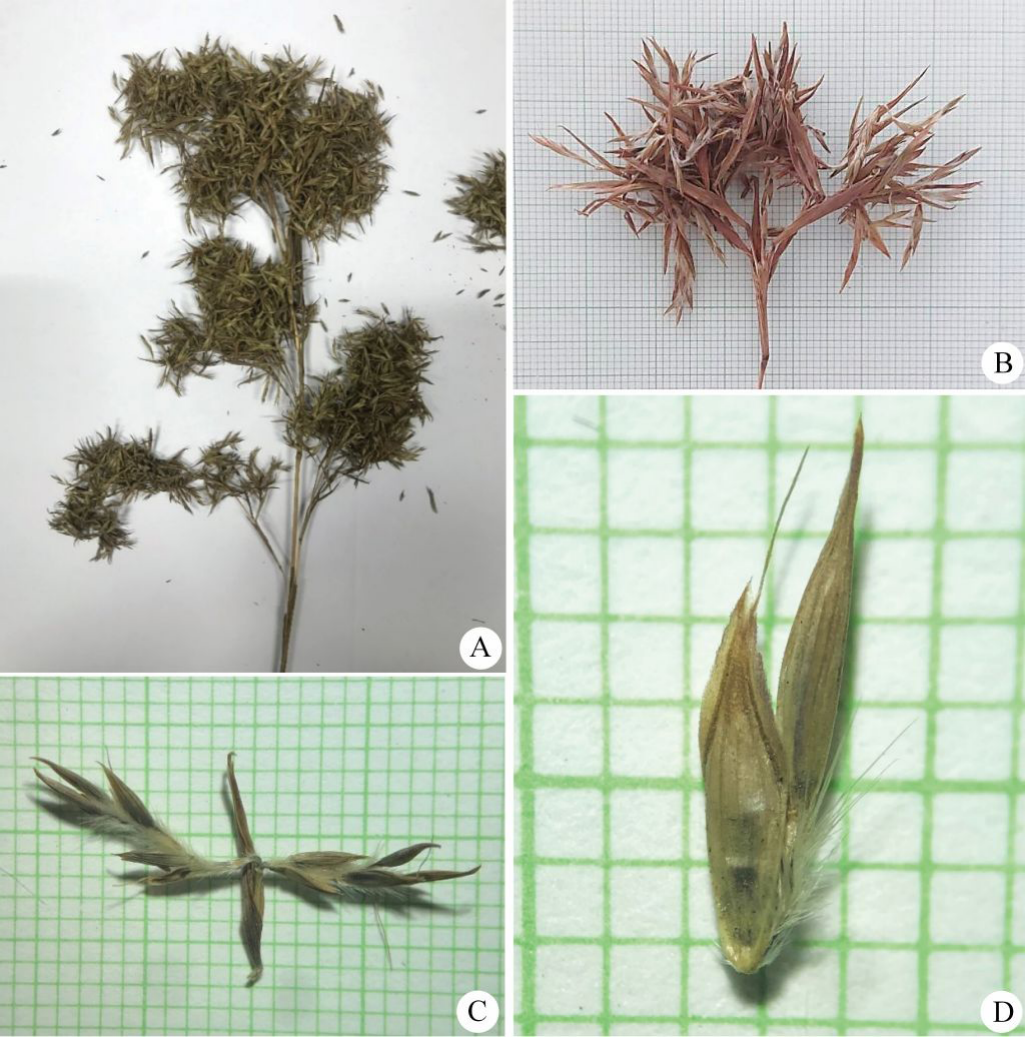
*Cymbopogon nardus*. (A) Synflorescence. (B) Part of synflorescence. (C) True inflorescence, which in this study refers specifically to a unit consisting of a pair of racemes subtended by a spatheole. (D) Paired spikelet. Photos: Paweena Wessapak.

Perennial, tufted. *Culms* erect, up to 2 m high (including synflorescences); nodes glabrous, glabrescent or tomentose; internodes terete, 30–35 cm long, 4–6 mm diam., glabrous. *Leaf sheaths* 20–25 cm long, margins membranous and entire, glabrous. *Ligules* chartaceous, 3–4 mm long. *Collar* glabrescent or pubescent. *Leaf blades* linear, 90–120 × 1.5–2 cm, apex acute, base narrow or attenuate and slightly glabrous, margins scabrous, chartaceous or tough, glabrous on both surfaces. *Synflorescence* 65–80 × 20–25 cm, large panicle, much congested, often interrupted.; synflorescence a spatheate panicle composed of multiple true inflorescences; each true inflorescence consisting of a pair of spike-like racemes subtended by a spatheole. *Raceme* 2, subtended by spatheole 1.5–1.7 cm long, chartaceous, glabrous; lower raceme ca. 1 cm long, 4 spikelet pairs (including homogamous pairs); upper raceme 1.2(−1.5) cm long, 3 spikelet pairs; raceme-based slightly flattened, hairy; rachis internode flattened, hairy along margins and on the back. *Pedicel of homogamous pair* not swollen. *Peduncle* terete, 5–7 mm long, glabrous. *Spikelet* in pair or triad. *Sessile spikelets* lanceolate, 4–4.5 × ca. 1 mm. *Lower glume* lanceolate, 4–4.5 × ca. 1 mm, apex bifid, narrowly or slightly broadly winged, margins entire, chartaceous, flat or slightly concave at the base, glabrous, obscurely 3–4(–5)-nerved. *Upper glume* lanceolate, boat-shaped, 4–4.2 × ca. 0.6 mm, apex acuminate, margins ciliate, chartaceous, 1-keeled with narrowly winged, glabrous, 1-nerved. *Florets* 2. *Lower floret* sterile. *Lower lemma* elliptic or lanceolate, ca. 3.5 × 0.6 mm, apex acute, margins ciliate, hyaline, glabrous, nerveless. *Lower palea* absent. *Upper floret* bisexual. *Upper lemma* ca. two mm long, apex bifid, shortly awn from sinus; awn geniculate, 4–9 mm long (shortly exsert) or awnless. *Upper palea* absent. *Lodicules* cuneate, ca. 0.4 mm long, truncate. *Stamens*: filament filiform; anther brown, 1.5–2 mm long. *Pistil*: ovary lanceolate in outline, ca. 1 × 0.2 mm; style 2; stigma plumose. *Caryopsis* not seen. *Pedicelled spikelet* lanceolate or narrowly elliptic, 3.3–4.2 × 0.8–1 mm. *Pedicel* flattened, 2–2.5 mm long, hairy along margins and glabrous on the back. *Lower glume* lanceolate or narrowly elliptic, 3.3–4.2 × ca. 0.8 mm, apex acute or acuminate, margins entire, chartaceous, glabrous, 7-, 9-, or 11-nerved. *Upper glume* lanceolate, 3–4.2 × 0.6–0.8 mm, apex acute, margins ciliate, chartaceous, glabrous, 3-nerved. *Lower lemma* oblong, ca. 3 × 0.5 mm, apex acute, margins ciliate, hyaline, glabrous, nerveless. (Fig. 15)

**Distribution.** India, Sri Lanka, and Indonesia.

**Distribution in Thailand.** Cultivated throughout the country.

**Phenology.** Flowering and fruiting from October to March.

**Vernacular names.** Cha khai ma khut ( 
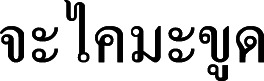
) (Northern); Ta khrai daeng ( 
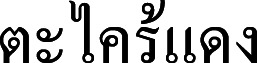
) (Nakhon Si Thammarat); Ta khrai ma khut ( 
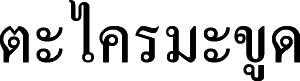
) (Northern); **Ta khrai hom** (**
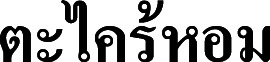
**) (Central); Ceylon citronella, Citronella grass (English).

**Uses.** It is cultivated for the production of citronella oil.

**Lectotypification.** *Andropogon confertiflorus* was published by Steudel (1854) with the type locality given simply as “Nilagiri”. The name was later treated as *Cymbopogon nardus* var. *confertiflorus* by *Bor (1953)*. Steudel neither designated a type specimen nor provided details of the collector or herbarium. Multiple specimens collected by *R. F. Hohenacker 932* from the Nilagiri have been located in several herbaria, namely BM [BM00959804], E [E00393597], FI [FI012282], K [K000245868], P [P00745754], and S [S-G-402]. As these specimens constitute original material and no type specimen was designated in the protologue, they are syntypes under Art. 9.6 of the ICN (Turland et al., 2018). The specimen at K [K000245868], which is the most complete and best preserved, is designated here as the lectotype; all remaining specimens are designated as isolectotypes, in accordance with Arts. 9.3 and 9.12 of the ICN (Turland et al., 2018).

**Specimens examined.** **North-Eastern**: Udon Thani [Cultivated, Kumphawapi, 4 Dec 2008, *M. Norsaengsri 4578* (QBG)]; **Eastern**: Nakhon Ratchasima [Cultivated, Sakaerat, 8 Mar 2018, *P. Wessapak 448* (BK)]; **Peninsular**: Satun [Tarutao, 22 Oct 1979, *G. Congdon 70* (AAU)]; **Unknown Locality**: [*T. Smitinand 2064* (BKF072171)].

**11. *Cymbopogon traninhensis*** (A. Camus) Soenarko, Reinwardtia 9: 347. 1977.

≡ *Cymbopogon confertiflorus* (Steud.) Stapf var. *traninhensis* A. Camus, Bull. Mus. Natl. Hist. Nat. 26: 565. 1920; A. Camus in Lecomte, Fl. Indo-Chine 7: 341. 1922. Type: Laos, Traninh, 1919, *R. Miéville s.n.* (holotype: **P**! [P00745758]).

= *Cymbopogon khasianus* (Hack.) Stapf ex Bor var. *nagensis* Bor, J. Bombay Nat. Hist. Soc. 52: 169. 1954. Type: India, Assam, Naga Hills, Shiloi Jopi, 16 Nov 1935, *N. L. Bor 10* (holotype: **K**! [K000245865]).

**Figure 16:**
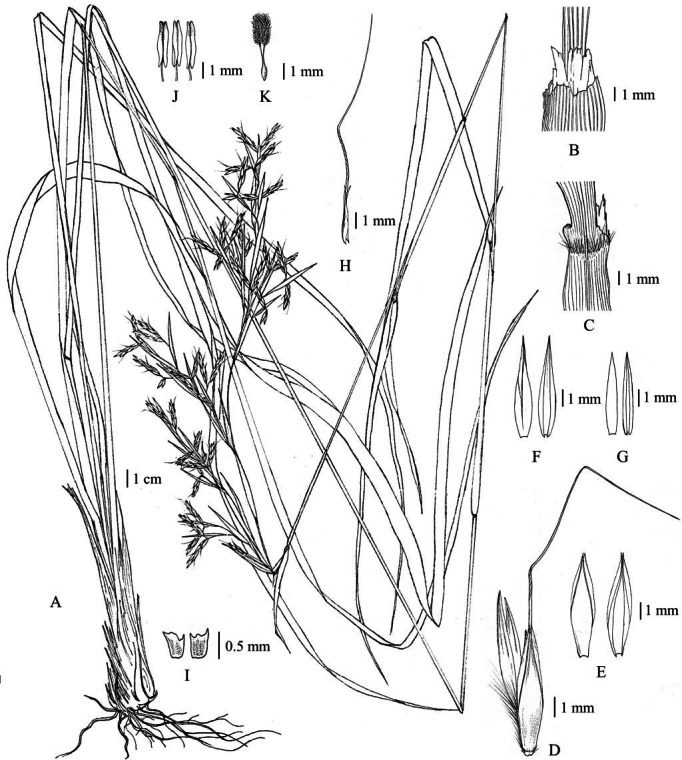
*Cymbopogon traninhensis*. (A) Habit. (B) Ligule. (C) Collar. (D) Paired spikelet. (E) Lower glumes. (F) Upper glumes. (G) Lower lemmas. (H) Upper lemma with distinct awn. (I) Lodicules. (J) Stamens. (K) Pistil. Photo: Drawn by Paweena Wessapak.

**Figure 17:**
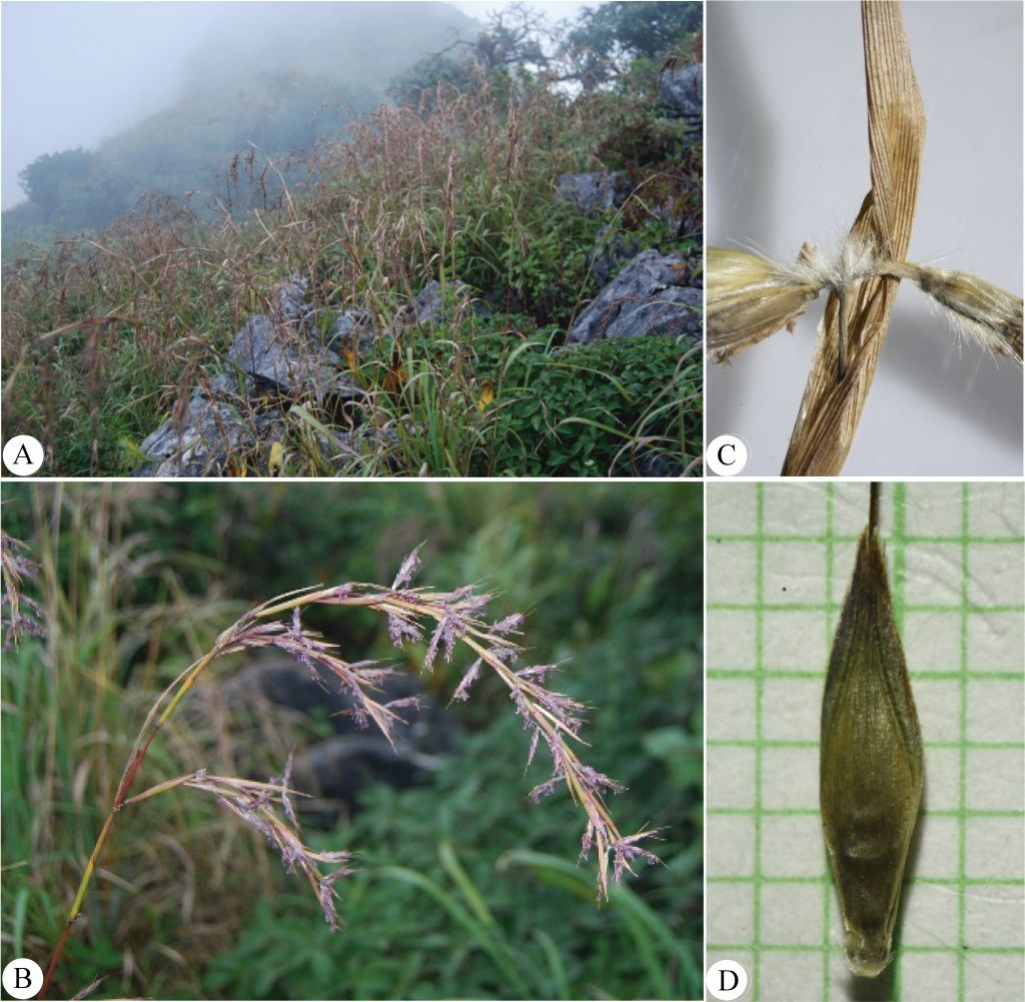
*Cymbopogon traninhensis*. (A) Habitat and habit. (B) Synflorescence. (C) Raceme-based with spatheole. (D) Sessile spikelet. Photos: Paweena Wessapak.

Perennial, tufted. *Culms* erect, 0.8–1.6 m high (including synflorescences); nodes pubescent or glabrescent; internodes terete, 15–30 cm long, 1.5–5.5 mm diam., glabrous. *Leaf sheaths* 9–28 cm long, margins membranous, glabrous. *Ligules* chartaceous, 4–6 mm long. *Collar* hairy. *Leaf blades* linear, 22–80 × 0.8–1.3(−1.5) cm, apex acute, based narrow, margins scabrous, chartaceous, glabrous on both surfaces. *Synflorescence* 20–80 × 8–20 cm.; synflorescence a spatheate panicle composed of multiple true inflorescences; each true inflorescence consisting of a pair of spike-like racemes subtended by a spatheole. *Racemes* 2, subtended by spatheole 2.2–3.5 cm long, chartaceous, glabrous; lower raceme 1.4–2.3 cm long, (3–)4–5 spikelet pairs (including homogamous pair); upper raceme 1.7–2.6 cm long, (3–)4–6 spikelet pairs; raceme-based flattened, hairy; rachis internode flattened, hairy along margins, glabrous on the back. *Pedicel of homogamous pair* not swollen. *Peduncle* terete, 0.5–1.5 cm long, glabrous. *Spikelet* in pair or triad. *Sessile spikelets* lanceolate or broadly lanceolate, narrow at base, (6–)6.5–8.5 × 1–1.8 mm. *Lower glume* lanceolate or broadly lanceolate, (6–)6.5–8.5 × 1–1.8 mm, apex bifid, broadly winged, margins entire, flat or shallowly concave below the middle, 1–3-wrinkled, glabrous, 2-nerved or nerveless. *Upper glume* boat-shaped, 5.5–7.5 × ca. 1 mm, apex acuminate, margins ciliate, chartaceous, 1-keeled with winged on keel, 1- or 3-nerved. *Florets* 2. *Lower floret* sterile. *Lower lemma* oblong-lanceolate, 4.8–6 × 0.6–1 mm, apex acute or acuminate, margins ciliate, membranous, glabrous, 2-nerved. *Lower palea* absent. *Upper floret* bisexual. *Upper lemma* 3.8–5.5 mm long, apex bifid, awn from sinus; awn geniculate, 1.3–1.8 cm long, scabrous. *Upper palea* absent. *Lodicules* cuneate, ca. 0.5 mm long, truncate. *Stamens*: filament filiform; anther yellow, 2.5–3 mm long. *Pistil*: ovary lanceolate in outline, 1–1.3 × ca. 0.3 mm; style 2; stigma plumose. *Caryopsis* not seen. *Pedicelled spikelets* lanceolate, 5–7 × 1–1.5 mm, *Pedicel* flattened, 2.5–3 mm long, hairy along margins and on both sides or glabrous on the back. *Lower glume* lanceolate, 5–6.7 × 1–1.2 mm, apex bifid, narrowly winged, margins ciliate, chartaceous, glabrous, 9- or 13-nerved. *Upper glume* lanceolate, 5–6.2 × 0.8–1 mm, apex acuminate, margins ciliate, subchartaceous, glabrous, 3-nerved. *Lower lemma* oblong-lanceolate, 4–5.8 × 0.6–1 mm, apex acuminate, margins ciliate, membranous, glabrous, 1-nerved or nerveless (Figs. 16 & 17).

**Distribution.** India (Assam), Myanmar, China (Yunnan), Laos, and Thailand.

**Distribution in Thailand.** **Northern**: Chiang Mai.

**Habitat and Ecology.** Rocky slope areas, open on granite or limestone hills, scattered in lower montane forests; 400−2,000 m alt.

**Phenology.** Flowering and fruiting from September to December.

**Vernacular name.** **Ta khrai pa cho dok muang** (**
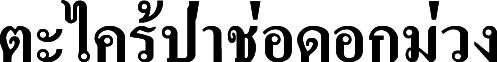
**), proposed here.

**Uses.** Not reported.

**Notes.** The specific characters are spikelets more than six mm long, broadly winged and narrowing downward. The synflorescence is usually dark purplish, although some specimens from Yunnan may be yellowish-green (Soenarko, 1977).

**Specimens examined.** **Northern**: Chiang Mai [Doi Chiang Dao, 1,900 m alt., 7 Dec 1959, *T. Smitinand & E. G. Abbe 6263* (BKF, K); ibid., 1,600–1,900 m alt., 2 Dec 1961, *T. Smitinand & J. A. R. Anderson 7258* (K); ibid., 1,600–1,850 m alt., 11 Nov 1962, *T. Smitinand 7838* (BKF, K); Chiang Dao, 24 Oct 2015, *P. Wessapak, W. Aiyakool, K. Kommongkol, T. Napiroon, W. Salee, S. Sarapol & K. Sisakhon 266, 268* (BK); Doi Mon Luang, Pong Yaeng, Mae Rim, 1,500 m alt., 1 Sep 1994, *W. Nanakorn et al. 2281* (QBG); Mae Taeng, 400 m alt., 6 Oct 1994, *W. Nanakorn et al. 2287* (QBG)].

**12. *Cymbopogon winterianus*** Jowitt ex Bor, Oesterr. Bot. Z. 12: 185. 1965; Bor, J. Bombay Nat. Hist. Soc. 52: 906. 1953; Backer & Bakh. f., Fl. Java 3: 611. 1968. Type: Sri Lanka, Pillagoda Valley, Baddegama, 11 Feb 1908, *A. W. Winter s.n.* (lectotype, designated by Bor (1965): **K**! [K000245866]).

**Figure 18:**
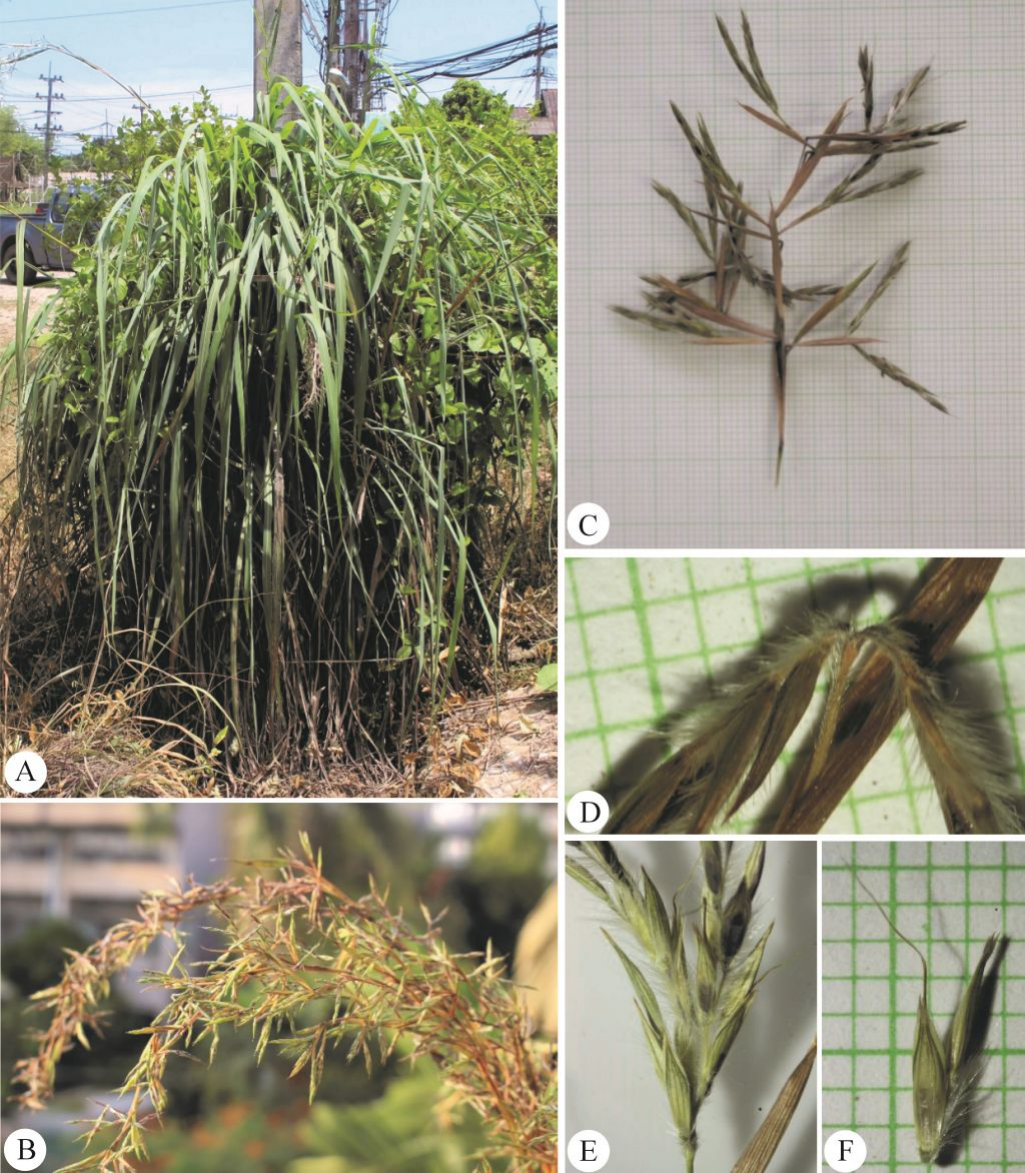
*Cymbopogon winterianus*. (A) Habitat and habit. (B–C) Parts of synflorescences. (D) Raceme-based with spatheole. (E) Base of racemes. (F) Paired spikelet. Photos: Paweena Wessapak.

Perennial, tufted. *Culms* erect, 1.8–2.3 cm high (including synflorescences); nodes glabrous; internodes 12–26 cm long, 2–7 mm diam., glabrous. *Leaf sheaths* 18–37 cm long, glabrous. *Ligules* membranous or chartaceous, 3.5–5.5 mm long. *Collar* glabrous or slightly bearded. *Leaf blades* linear, 90–100 × 1.3–1.6 cm, apex acute, base narrow or attenuate, sometimes pubescent, margins scabrous, chartaceous, both surfaces glabrous. *Synflorescence* 80–100 × 30–50 cm, lax.; synflorescence a spatheate panicle composed of multiple true inflorescences; each true inflorescence consisting of a pair of spike-like racemes subtended by a spatheole. *Racemes* 2, arranged along a zig-zag axis, subtended by spatheole 1.3–2.4 cm long, chartaceous, glabrous; lower raceme 1.2–2 cm long, 3–5 spikelet pairs (including homogamous pair); upper raceme 1.4–2.2 cm long, 3–5 spikelet pairs; raceme-based slightly flattened or terete, hairy; rachis internode flattened, hairy along margins. *Pedicel of homogamous spikelet* not swollen. *Peduncle* terete, 0.6–1 cm long, glabrous. *Spikelet* in pair or triad. *Sessile spikelets* elliptic-lanceolate or broadly lanceolate, 3.5–4.8 × 0.8–1.2 mm. *Lower glume* elliptic-lanceolate or broadly lanceolate, 3.5–4.8 × 0.8–1.2 mm, apex bifid, narrowly winged, chartaceous, slightly concave below the middle, with or without 2-wrinkled, glabrous, 3–5-nerved. *Upper glume* boat-shaped, 3.3–4 × ca. 0.8 mm, apex acuminate, margins ciliate, chartaceous, glabrous, 1-nerved. *Florets* 2. *Lower floret* sterile. *Lower lemma* lanceolate or oblong, 3–4 × 0.5–0.7 mm, apex acuminate, margins ciliate, hyaline or membranous, glabrous, nerveless. *Lower palea* absent. *Upper floret* bisexual. *Upper lemma* 2.2–3 mm long, apex bifid, short awn or bristle from sinus; awn 1–5 mm long, rarely up to eight mm long (not or shortly exserted) or awnless. *Upper palea* absent. *Lodicules* cuneate, ca. 0.4 mm long, truncate. *Stamens*: anther yellow, 1–2 mm long. *Pistil*: ovary oblong or lanceolate in outline, 0.5–0.7 mm long; style 2; stigma plumose. *Caryopsis* not seen. *Pedicelled spikelet* lanceolate, 3.5–4 × ca. 0.8 mm. *Pedicel* flattened, 2.5–3 mm long, hairy along margins and on the back. *Lower glume* lanceolate, 3.5–4 × ca. 0.8 mm, apex acuminate, chartaceous, glabrous, 9-nerved. *Upper glume* narrowly elliptic, 3–4 × 0.6–0.8 mm, apex acute, margins ciliate, chartaceous or membranous, glabrous, 3-nerved. *Lower lemma* oblanceolate or elliptic, 2.5–3.2 × 0.4–0.6 mm, apex acuminate, margins ciliate, hyaline, glabrous, nerveless (Fig. 18).

**Distribution.** Sumatra, Java, and Borneo.

**Distribution in Thailand.** Cultivated throughout the country.

**Phenology.** Flowering and fruiting from December to April.

**Vernacular names.** **Ta khrai** **hom** (**
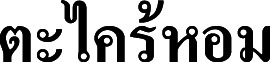
**) (General); Citronella grass, Java Citronella (English).

**Uses.** It is cultivated for the production of citronella oil.

### Specimens examined

**Northern**: Chiang Mai [Cultivated, Chiang Mai University, 22 Dec 1990, *J. F. Maxwell 90-1367* (AAU)]; **South-Western**: Kanchanaburi [Cultivated, Nong Rong, Phanom Thuan District, 43 m alt., 26 Mar 2017, *P. Wessapak 357* (BK)]; **Central**: Bangkok [Cultivated, 30 Dec 1931, *A. F. G. Kerr 20648* (K)]; **South-Eastern**: Rayong [Cultivated, Ban Na, Klaeng, 30 Apr 2017, *P. Wessapak 366* (BK)]; Chanthaburi [Ban Ang, Makham, 8 Dec 1945, *C. Nupakdee 171* (BKF)]; Trat [Cultivated, Ko Kut, 2 m alt., 6 Apr 1959, *T. Smitinand 5708* (BKF, K)]; **Peninsular**: Surat Thani [Thung Song, 14 Feb 1929, *Put 2392* (BK, BM, K); Narathiwat [Cultivated, 11 Feb 2017, *C. Ngernsaengsaruay, N. Meeprom & A. Thonglim s.n.* (BK)].

## Discussion

Camus (1919) treated *C. annamensis* as a variety of *C. martinii*. The principal morphological difference between the two taxa lies in the shape of the leaf blade base: in typical *C. martinii* it is cordate, whereas in *C. annamensis* it is rounded to attenuate. In the following year, Camus (1920) raised the taxon to the rank of a distinct species. *Cymbopogon annamensis* is distributed from China to Indo-China, whereas the distribution of *C. martinii* ranges from the Himalaya to Vietnam and is widely cultivated in several countries for the production of aromatic oil. To date, there are no reports of the utilization of *C. annamensis* as a source of essential oil.

*Cymbopogon calciphilus* is similar to *C. flexuosus* in having a narrowly winged lower glume of the sessile spikelet, but differs by its narrower synflorescence and leaf blade. The number of nerves on the lower glume of sessile spikelets is often variable, being either nerveless or 2–3-nerved (Soenarko, 1977). Specimens in Thailand may reach 1–1.5 m, occasionally up to 1.8 m, although the species is usually less than 1.2 m tall. Diagnostic characters include a narrowly synflorescence, narrowly winged lower glume, basal sheaths, triangular patches, and hairy nodes.

*Cymbopogon cambogiensis* is recognized by the lowermost pedicel being somewhat flattened and the lower glume of the sessile spikelet with a shallow median groove. Sessile spikelets of specimens from Thailand are generally smaller (2.3–3.2 × 0.7–1 mm) than those cited by Soenarko (1977) (3–3.2 mm long). It usually resembles *C. mekongensis* in having a lower glume with a median groove and a lowermost pedicel somewhat flattened. Although ligule length was previously noted as differing (ca. five mm in *C. cambogiensis vs.* ca. 1 mm in *C. mekongensis*), our examination of the type and additional material indicates that ligule length overlaps (1–2 mm) in the specimens examined, and therefore ligule length alone is not reliable for distinguishing these two species. Identification should instead focus on sessile spikelet size, with *C. cambogiensis* generally having smaller and shorter sessile spikelets than *C. mekongensis*. In addition, *C. mekongensis* typically has a narrow leaf blade base, whereas *C. cambogiensis* has a leaf blade base rounded or subcordate.

*Cymbopogon travancorensis* was originally described as a distinct species by Bor (1954), but subsequent taxonomic treatments have placed this name as a heterotypic synonym of *C. flexuosus* According to major taxonomic backbones and synonymy databases (*e.g.*, Kew’s Plants of the World Online, World Grass Species), *C. travancorensis* is accepted as synonymous with *C. flexuosus* because it falls within the morphological and nomenclatural circumscription of *C. flexuosus*, and multiple names published for related material have been consolidated under *C. flexuosus* as the accepted name. Therefore, we follow this widely accepted treatment and include *C. travancorensis* in the synonymy of *C. flexuosus* in the present work.

*Cymbopogon khasianus* exhibits considerable morphological variation. It resembles *C. traninhensis* in having slender racemes and a lower glume of the sessile spikelet with wings that are expanded above the middle and tapering toward the base. Chen & Phillips (2006) reported that specimens of *C. khasianus* from China have a purplish panicle, whereas Soenarko (1977) noted that *C. traninhensis* from Yunnan has a yellowish panicle. In the present study, panicles of *C. khasianus* were usually observed as green to yellowish green at comparable stages of development.

*Cymbopogon khasianus* is reliably distinguished from *C. traninhensis* by its shorter sessile spikelets (about 4–5 mm long in *C. khasianus* according to World Flora Online data). The lower glume of the sessile spikelet in *C. khasianus* typically bears four to five nerves, although these may be obscure in some specimens. Comparable data for the number of veins in *C. traninhensis* is not consistently reported, but the combination of sessile spikelet size, glume winging, and panicle colour and form supports separation of these two species in this study.

In Thailand, *C. martini* was previously recorded as an exotic grass (Pooma & Suddee, 2014). However, examination of herbarium material indicates that it occurs in natural habitats. Specimens document its presence in open deciduous forest (*A. F. G. Kerr 10192*), on hanging roadbanks (*S. Laegaard & M. Norsaengsri 21786*), on limestone hills across the Khwae River from Khao Salop National Park (*M. Lazarides 7422*), and gregariously on white sand at the base of granite hills (*M. Lazarides 7448*). The species is native to the Himalaya, Pakistan, India, Nepal, Bangladesh, Myanmar, and Vietnam (POWO, 2025). Considering its distribution in neighbouring countries and repeated records from wild habitats in Thailand, this study suggests that the species should be considered native to Thailand.

*Cymbopogon clandestinus* (Nees ex Steud.) Stapf, a name reported in some Thai literature (Nanakorn, 1990; Nanakorn & Norsangsri, 2001), has no specimens recorded in Thailand in the present study. The only specimen previously cited for this species (BK33204) was identified as *C. martini* in our study.

*Cymbopogon khasianus* var. *nagensis*, a synonym of *C. traninhensis*, was described by Bor (1954) as having a pubescent lower glume on the sessile spikelet. However, examination of the type specimen of *C. traninhensis* shows that the lower glume is glabrous, similar to specimens from Thailand.

*Cymbopogon winterianus* is often confused with *C. nardus*. Both produce citronella oil from their leaves, but *C. winterianus* is native to West Malesia and mainly cultivated in Indonesia (Java citronella), whereas *C. nardus* is preferred in Sri Lanka (Ceylon citronella).

*Cymbopogon winterianus* can be distinguished from *C. nardus* by the more distinct nerves on the lower glume and generally broader, shorter leaf blades (Bor, 1953). Soenarko (1977) reported similar leaf width, which is consistent with the present study. The synflorescence of *C. winterianus* is much laxer and more effuse than that of *C. nardus*, and its peduncle is usually longer, in agreement with our observations.

## Conclusions

This taxonomic revision provides a comprehensive and up-to-date account of the genus *Cymbopogon* (Poaceae) in Thailand. Twelve species are recognized, including one endemic species, *C. calciphilus*. Detailed morphological examination of extensive herbarium material and field observations has clarified species delimitations. Three names, *C. cambogiensis*, *C. khasianus*, and *C. confertiflorus* (a synonym of *C. nardus*) are lectotypified here to stabilize nomenclature and ensure consistent application of names. Diagnostic characters of taxonomic importance include features of the synflorescence structure, raceme arrangement, spikelet morphology, glume wings and nervation, and awn development, which together allow reliable species identification.

Among the twelve species treated, *C. citratus*, *C. nardus*, and *C. winterianus* are widely cultivated in Thailand for their economically valuable aromatic oils but show no evidence of naturalization. In contrast, *C. martini*, previously regarded as exotic, is here considered native based on repeated collections from natural habitats and its distribution in neighboring regions. Updated information on distribution, habitat, phenology, vernacular names, and uses is provided for all species, contributing to a more accurate understanding of their ecological and economic significance.

This study provides a robust taxonomic framework for *Cymbopogon* in Thailand and serves as a reliable reference for future floristic, ecological, and applied research, as well as for conservation planning and sustainable utilization of aromatic grass resources.

## Supplemental Information

10.7717/peerj.21297/supp-1 Supplemental Information 1 Raw Data The additional specimens examined.
The numerical values reported in the manuscript represent direct morphological measurements (e.g., length and width) of the examined specimens. These data were not subjected to statistical analysis and are provided solely to describe the observed size ranges.

## Additional Information and Declarations

### Field Study Permissions

The following information was supplied relating to field study approvals (i.e., approving body and any reference numbers): Additional plant specimens were collected outside protected areas, where no specific permits were required. The study was based on collected material and the examination of specimens housed in the following herbaria: AAU, BK, BKF, BM, C, K, P, PSU, and QBG.

### Data Availability

The following information was supplied regarding data availability: The raw data (additional specimens examined) is available in the Supplementary Files.
