# Supplementary material for: A taxonomic revision of Cymbopogon (Poaceae) in Thailand
Source: PeerJ. 2026 May 18;14:e21297. doi: 10.7717/peerj.21297 (PMC13192463; doi:10.7717/peerj.21297)
Supplement: Supplemental Information 1 — The additional specimens examined. The numerical values reported in the manuscript represent direct morphological measurements (e.g., length and width) of the examined specimens. These data were not subjected to statistical analysis and are provided solely to describe the observed size ranges. [file peerj-14-21297-s001.docx]

Raw Data

The raw data is available in the additional specimens examined.

**1.** ***Cymbopogon annamensis*** (A. Camus) A. Camus

**Specimens examined.** **Northern**: Phetchabun [Thung Salaeng Luang, 600 m alt., 27 Aug 2017, *P. Wessapak 383* (BK); ibid., 600 m alt., 30 Sep 2017, *P. Wessapak 402* (BK)]; ibid., 12 Oct 2018, *P. Wessapak, W. Arthan & J.* *Satthaphorn 479* (BK)]; **Eastern**: Chaiyaphum [Phu Khiao Wildlife Sanctuary, 300 m alt., 11 Nov 1984, *W. Nanakorn et al. 2084* (QBG)]; Nakhon Ratchasima [Locality unspecified, 23 Nov 1923, *A. F. G. Kerr 7948* (BK, K); Pak Thong Chai, 200 m alt., 27 Dec 1923, *A. F. G. Kerr 8130* (AAU, BK, BM, K); Bua Yai, 1 Nov 1931, *Put 4245* (BM, BK, K)]*.*

**2. *Cymbopogon calcicola*** C. E. Hubb.

**Specimens examined.** **Peninsular**: Krabi [Ko Phi Phi, 9 Apr 1930, *A. F. G. Kerr 18895* (BKF, BM, K)]; Pangnga [near Ko Pan Yi, 16 Dec 1918, *Md. Haniff & Nur 4074* (K, SING)]; Trang [Hat Ratchamongkhon, 16 Mar 2018, *P. Wessapak, C. Ngernsaengsaruay, N. Meeprom & W. Boonthasak 454* (BK)].

**3.** ***Cymbopogon calciphilus*** Bor

**Specimens examined.** **Northern**: Lampang [Hill between Thoen and Li, 600 m alt., 29 Nov 1959, *T. Smitinand & E. C. Abbe 6165* (BKF, K)]; Sukhothai [Locality unspecified, 4 Nov 1971, *J. F. Maxwell 71-693* (AAU)]; **Eastern**: Nakhon Ratchasima [Lat Bua Khao, 7 Nov 1931, *Put 4319* (BK, BM, K)]; Ubon Ratchathani [Phu Chong Na Yoi, 230 m alt., 22 Oct 2017, *P. Wessapak 417* (BK)]; **North-Eastern**: Loei [Phu Kradueng, Sam Haek, 400 m alt., 16 Oct 1954, *T. Smitinand 2028* (BKF, K), ibid, 18 Nov 2017, *P. Wessapak 437, 438* (BK)]; Sakhon Nakhon [Phu Phan, 250 m alt., 8 Oct 2017*, P. Wessapak, C. Ngernsaengsaruay, R. Meeboonya, N. Meeprom, W. Boonthasak, T. Ausadamongkol & K. Kampeera 407* (BK)]; Khon Kaen [Phu Wiang, 300 m alt., 5 Feb 1931, *A. F. G. Kerr 19998* (BK, BM, K), ibid., 300 m alt., 28 May 2016, *P. Wessapak, R. Meeboonya & P. Yodboplub 315* (BK), ibid., 29 Oct 2017, *P. Wessapak 422* (BK); **South-Western**: Kanchanaburi [Erawan National Park, 18 Nov 1970, *M. Lazarides 7424* (C, K); Hin Dat, 50 m alt., 26 Nov 1957, *T. Smitinand 3870* (K); Locality unspecified, 14 Dec 1930, 50 m alt., *A. F. G. Kerr 19876* (BK, BM, K)]; Phetchaburi [Thung Luang, 100 m alt., 8 Nov 1931, *A. F. G. Kerr 20596* (BK, BM, K)]; Prachuap Khiri Khan [Hua Hin, 10 m alt., 6 Nov 1927, *A. F. G. Kerr 13478* (BKF, BM, K); Khao Tao, 20 m alt., 9 Nov 1928, *A. Marcan 2437* (BM, K)].

**4.** ***Cymbopogon cambogiensis*** (Balansa) E. G. Camus & A. Camus

**Specimens examined.** **Northern**: Sukhothai [Si Satchanalai, 18 Jul 2015, *P. Wessapak, C. Ngernsaengsaruay, S. Chodchoy, N. Meeprom 248* (BK)]; Phitsanulok [Tha Pho, 300 m alt., 27 Dec 1961, *K. Larsen 8989* (C, K)]; **Central**: Chai Nat [Manorom, under 50 m alt., 19 Sep 1930, *A. F. G. Kerr 19677* (BK, BM, K)]; Suphan Buri [Bang Pla Ma, 22 Sep 1930, *A. F. G. Kerr* *s.n.* (K)]; **South-Eastern**: Sa Kaeo [Aranyaprathet, 50 m alt., 9 Aug 1930, *A. F. G. Kerr 19586* (BK, BM, K)]; Chanthaburi [Makham, 50 m alt., 13 Jun 1963, *K. Larsen 10027* (BKF, C, K)]; **Peninsular**: Chum Phon [Ban Pak Khlong, 20 m alt., 12 Jan 1927, *A. F. G. Kerr 11391* (BK, BM, K)].

**5.** ***Cymbopogon citratus*** (DC.) Stapf,

**Specimens examined.** **Peninsular**: Pattani [Nong Chik, 19 Jan 1998, *Y. Sutdhikaran 7* (PSU)].

**6. *Cymbopogon flexuosus*** (Nees ex Steud.) Will. Watson

**Specimens examined.** **Northern**: Chiang Mai [Doi Saket, 1,100 m alt., 10 Jan 1969, *T. Smitinand & G. S. 10629* (BKF)]; **Central**: Bangkok [Cultivated, Kasetsart University, 13 Feb 2017, *P. Wessapak 353* (BK)]; **South-Eastern**: Chon Buri [Cultivated, Si Racha, 31 Mar 1923, *A. F. G. Kerr 6956* (BK, BM, K); Si Racha, 24 m alt., 22 Nov 1927, *D. J. Collins 1892* (BK, K)]; **Peninsular**: Satun [Ko Batuang, 50 m alt., 13 Jan 1928, *A. F. G. Kerr 14057* (BK, BM, K)].

**7. *Cymbopogon khasianus*** (Hack.) Stapf ex Bor

**Specimens examined.** **Northern**: Chiang Mai [Doi Suthep, 762 m alt., 12 Nov 1911, *A. F. G. Kerr 1554B* (BM, K); Mae Rim, 400 m alt., 23 Nov 1995, *W. Nanakorn et al. 5327* (QBG); Tha Pha, Mae Chaem, 1,170 m alt, 25 Nov 2017, *P. Wessapak 443, 444* (BK)].

**8. *Cymbopogon martini*** (Roxb.) Will. Watson

**Specimens examined.** **Northern**: Chiang Mai [300 m alt., 3 Nov 1960, *Anonymous s.n.* (BK33204)]; **North-Eastern**: Phetchabun [At km 25 on road 12 Lom Sak-Khon Kaen, 625 m alt., 25 Oct 2001, *S. Laegaard & M. Norsaengsri 21786* (AAU, QBG)]; **South-Western**: Kanchanaburi [Si Sawat, 100 m alt., 11 Jan 1926, *A. F. G. Kerr 10192* (BM, BK, K); Khao Salop National Park, 18 Nov 1970, *M. Lazarides 7422* (K)]; **South-Eastern**: Chon Buri [Locality unspecified, 24 Nov 1970, *M. Lazarides 7448* (K)].

**9. *Cymbopogon microstachys*** (Hook. f.) Soenarko

**Specimens examined. Northern**: Phetchabun [Nam Nao, 870 m alt., 30 Sep 2017, *P. Wessapak 403* (BK)]; **Central**: Lop Buri [Chai Badan, 100 m alt., 16 Dec 1923, *A. F. G. Kerr 8013* (BK, BM, K)]; **South-Western**: Kanchanaburi [Kaeng Pralom, 400 m alt., 25 Dec 1961. *K. Larsen 8939* (C, K)]; **Peninsular**: Surat Thani [Ko Phangan, 300 m alt., 23 Sep 1928, *A. F. G. Kerr 16074* (BK, BM, K); ibid., 17 Sep 2016, *P. Wessapak, C. Ngernsaengsaruay, R. Meeboonya & N. Meeprom 333, 334, 335* (BK)]; Krabi [Ko Lanta Yai, 12 Nov 1966, 20 m alt., *B. Hansen & T. Smitinanad 12252* (BKF, C, K, SING); ibid., 16 Mar 2018, *P. Wessapak, C. Ngernsaengsaruay, N. Meeprom & W. Boonthasak 449, 450, 452, 453* (BK)].

**10.** ***Cymbopogon nardus*** (L.) Randle

**Specimens examined.** **North-Eastern**: Udon Thani [Cultivated, Kumphawapi, 4 Dec 2008, *M. Norsaengsri 4578* (QBG)]; **Eastern**: Nakhon Ratchasima [Cultivated, Sakaerat, 8 Mar 2018, *P. Wessapak 448* (BK)]; **Peninsular**: Satun [Tarutao, 22 Oct 1979, *G. Congdon 70* (AAU)]; **Unknown Locality**: [*T. Smitinand 2064* (BKF072171)].

**11.** ***Cymbopogon traninhensis*** (A. Camus) Soenarko

**Specimens examined.** **Northern**: Chiang Mai [Doi Chiang Dao, 1,900 m alt., 7 Dec 1959, *T. Smitinand & E.G. Abbe 6263* (BKF, K); ibid., 1,600−1,900 m alt., 2 Dec 1961, *T. Smitinand & J. A. R. Anderson 7258* (K); ibid., 1,600−1,850 m alt., 11 Nov 1962, *T. Smitinand 7838* (BKF, K); Chiang Dao, 24 Oct 2015, *P. Wessapak, W. Aiyakool, K. Kommongkol, T. Napiroon, W. Salee, S. Sarapol & K. Sisakhon 266, 268* (BK); Doi Mon Luang, Pong Yaeng, Mae Rim, 1,500 m alt., 1 Sep 1994, *W. Nanakorn et al. 2281* (QBG); Mae Taeng, 400 m alt., 6 Oct 1994, *W. Nanakorn et al. 2287* (QBG)].

**12. *Cymbopogon winterianus*** Jowitt ex Bor

**Specimens examined.** **Northern**: Chiang Mai [Cultivated, Chiang Mai University, 22 Dec 1990, *J. F. Maxwell 90-1367* (AAU)]; **South-Western**: Kanchanaburi [Cultivated, Nong Rong, Phanom Thuan District, 43 m alt., 26 Mar 2017, *P. Wessapak 357* (BK)]; **Central**: Bangkok [Cultivated, 30 Dec 1931, *A. F. G. Kerr 20648* (K)]; **South-Eastern**: Rayong [Cultivated, Ban Na, Klaeng, 30 Apr 2017, *P. Wessapak 366* (BK)]; Chanthaburi [Ban Ang, Makham, 8 Dec 1945, *C. Nupakdee 171* (BKF)]; Trat [Cultivated, Ko Kut, 2 m alt., 6 Apr 1959, *T. Smitinand 5708* (BKF, K)]; **Peninsular**: Surat Thani [Thung Song, 14 Feb 1929, *Put 2392* (BK, BM, K); Narathiwat [Cultivated, 11 Feb 2017, *C. Ngernsaengsaruay, N. Meeprom & A. Thonglim s.n.* (BK)]**.**
